# Enhanced replication of a contemporary avian influenza A H9N2 virus in human respiratory organoids

**DOI:** 10.1080/22221751.2025.2576574

**Published:** 2025-10-16

**Authors:** Lin-Lei Chen, Jonathan Daniel Ip, Wan-Mui Chan, Stephanie Joy-Ann Lam, Rhoda Cheuk-Ying Leung, Cyril Chik-Yan Yip, Xiaojuan Zhang, Allen Wing-Ho Chu, Hoi-Wah Tsoi, Aixin Li, Kwok-Hung Chan, Carol Ho-Yan Fong, Lei Wen, Jane Hau-Ching Poon, Janice Yee-Chi Lo, Kristine Shik Luk, Jasper Fuk-Woo Chan, Honglin Chen, Jie Zhou, Kwok-Yung Yuen, Mike Yat-Wah Kwan, Kelvin Kai-Wang To

**Affiliations:** a State Key Laboratory for Emerging Infectious Diseases, Carol Yu Centre for Infection, Department of Microbiology, School of Clinical Medicine, Li Ka Shing Faculty of Medicine, The University of Hong Kong, Pokfulam, Hong Kong Special Administrative Region, People’s Republic of China; b Centre for Virology, Vaccinology and Therapeutics, Hong Kong Science and Technology Park, Hong Kong Special Administrative Region, People’s Republic of China; c Department of Microbiology, Queen Mary Hospital, Pokfulam, Hong Kong Special Administrative Region, People’s Republic of China; d Department of Health, Centre for Health Protection, Hong Kong Special Administrative Region, People’s Republic of China; e Department of Pathology, Princess Margaret Hospital, Hong Kong Special Administrative Region, People’s Republic of China; f Department of Infectious Disease and Microbiology, The University of Hong Kong-Shenzhen Hospital, Shenzhen, Hong Kong Special Administrative Region, People’s Republic of China; g Pandemic Research Alliance Unit at the University of Hong Kong, Hong Kong Special Administrative Region, People's Republic of China; h Department of Pediatrics and Adolescent Medicine, Princess Margaret Hospital, Hong Kong Special Administrative Region, People’s Republic of China

**Keywords:** Avian influenza, H9N2, human organoids, intrahost evolution, human adaptive amino acid substitutions

## Abstract

H9N2 is currently the second most common avian influenza A virus subtype infecting humans. Monitoring viral phenotypic and genotypic adaptation to humans is crucial for risk assessment. Here, we compared the replication of an H9N2 human isolate collected in 2024 (A/HK/2346/2024) to a human isolate collected in 1999 (A/HK/1073/1999). In Madin Darby canine kidney (MDCK) cells, A/HK/2346/2024 and A/HK/1073/1999 replicated to 8 and 5 log10 plaque-forming units (PFU) per ml, respectively. In both human nasal and lung organoids, A/HK/2346/2024 replicated to 6 log10 PFU/ml, but A/HK/1073/1999 failed to replicate in either organoid. The infection rates of both ciliated and non-ciliated cells and the ratios of infected 2,6/2,3 cells were higher for A/HK/2346/2024 than A/HK/1073/1999. Apart from the mammalian adaptive substitutions that were present in the nasopharyngeal specimen collected on day 1 post-symptom onset (pso) (HA-D183N/D190 T/Q192R/Q226L; NA-del62-64; PB2-A588V/K702R; PB1-I368V; PA-K356R/S409N; M1-R95K), the mammalian-adaptive substitution PB2-D253N emerged *de novo* on day 7 pso. Analysis of all human (n = 96) and avian influenza (*n* = 14,762) H9N2 deposited at GISAID showed the dominance of several human-adaptive substitutions in H9N2 strains collected from humans in recent years. In summary, we demonstrated that a recent H9N2 virus is more adapted to humans, and is able to replicate to high titres in both upper and lower human respiratory tract which may confer higher person-to-person transmissibility and virulence. Our study underscores the importance of human organoid-based phenotypic monitoring and inter/intrahost genotypic monitoring for assessing the zoonotic risk of avian influenza viruses.

## Introduction

The avian influenza A H9N2 virus has been reported to cause sporadic human infections since the late 1990s [1, 2]. There have been an increasing number of human H9N2 cases in recent years. As of 6^th^ June 2025, a total of 168 human cases of H9N2 have been reported, and it is currently the second most prevalent avian influenza virus (AIV) subtype that causes human infections, after H5N1 [3]. Although H9N2 is classified as a “low pathogenic avian influenza” due to its low virulence in wild birds or poultry, it can cause severe disease in humans. Among the 77 laboratory-confirmed human H9N2 cases reported from the Western Pacific Region between November 2003 and July 2022, 5 (6.5%) had severe disease including 2 (2.6%) deaths [4, 5]. Most human H9N2 cases have been reported from China, with other cases reported from Southeast Asia, South Asia, and Africa [2, 3, 6]. Subclinical infection is common. In Hong Kong and Chinese Mainland, the seroprevalence of H9N2 among poultry workers was much higher than influenza A H5N1, H7N9 or other zoonotic influenza viruses [7, 8].

H9N2 has been relatively neglected when compared with H5N1 and H7N9. However, several characteristics make H9N2 a potential public health threat to humans. First, H9N2 contributes to the internal genes of most AIVs that cause severe human disease, including those related to H3N8 [9, 10], H5N1 [11], H5N6 [12], H7N4 [13], H7N9 [14, 15], H10N3 [16] and H10N8 [17]. Notably, reassortment between H9N2 and the widely circulating H5N1 2.3.4.4b clade has been documented [18]. Second, H9N2 has now replaced H5N6 and H7N9 as the most prevalent AIV subtype among chickens and ducks in China [19]. Third, the majority of H9N2 carries the HA Q226L, I155T and H183N substitutions and preferentially binds to human 2,6-linked sialic acid (SA) receptor [19], and the neuraminidase (NA) stalk deletion at residue 62–64 which can enhance virus entry into human cells [19, 20].

In February 2024, a case of human H9N2 infection was confirmed in Hong Kong. In view of the increasing number of human cases in recent years, we hypothesize that H9N2 has become better adapted to humans. To this end, we compared the replicative fitness of this 2024 H9N2 strain with a historical H9N2 strain collected in 1999, using the human nasal and lung organoid model [11, 21-23]. We also analysed serial specimens of this 2024 patient and determined the *de novo* adaptive substitutions that emerged during infection.

## Methods

The details of the methodologies are shown in the Supplementary Methods.

### Ethical approval

This study has been approved by the Institutional Review Board of the University of Hong Kong/Hospital Authority of Hong Kong West Cluster (UW 22-328 and UW 23-376) and the Central Institutional Review Board of the Hospital Authority of Hong Kong (CM20230814).

### Total nucleic acid extraction

Total nucleic acid (TNA) extraction was performed by the EZ1 Advanced XL instrument (QIAGEN, Hilden, Germany) using EZ1 Virus Mini Kit v2.0 (QIAGEN) according to manufacturer’s instructions.

### Influenza virus RT-PCR

One-step real-time reverse transcription-polymerase chain reaction (RT-PCR) assays for influenza virus detection were performed using QuantiNova Probe RT-PCR Kit (QIAGEN). The primers and probes used in this study are shown in Supplementary Table S1 [24, 25].

### Multiplex PCR

Multiplex PCR was performed by the in-house multiplex PCR at Public Health Laboratory Centre (PHLC) or the Avalon Automated Multiplex System (Emerging Viral Diagnostics [HK] limited) [26]. The pathogen targets of these multiplex PCR assays are shown in Supplementary Table S2.

### Metagenomic RNA sequencing

Metagenomic RNA sequencing was performed using the Oxford Nanopore MinION device (Oxford Nanopore Technologies, Oxford, United Kingdom). Library preparation using the sequence-independent single-primer amplification (SISPA) method was described previously [27].

### Whole genome sequencing and bioinformatics analysis for influenza virus

Influenza virus H9N2 whole genome sequencing was performed using the Oxford Nanopore MinION device (Oxford Nanopore Technologies, Oxford, United Kingdom). The consensus sequences were deposited into GISAID (Supplementary Table S3).

### Phylogenetic analysis

All segments of the consensus sequences were compared against the Influenza Nucleotide Database of GISAID using MEGABLAST with default parameters. The aligned nucleotide sequences were used to construct phylogenetics trees.

### Frequency of human adaptation substitutions in H9N2 sequences

Amino acid (aa) sequences of all 96 human (599 aa sequences) and 14,762 avian H9N2 (50,577 aa sequences) from GISAID were retrieved on 11^th^ February 2025.

### Protein–protein docking between H9N2 nucleoprotein (NP) and MX1

Full-length structures of mutant (R19C) H9N2 NP and human MX1 dimer were predicted with AlphaFold2 [28] and AlphaFold2-multimer [29], respectively. Protein–protein docking simulation was performed by using Rosetta high-resolution full-atom docking protocol [30].

### Viral culture from clinical specimens

Viral culture was performed on Madin Darby canine kidney (MDCK) cells in the Biosafety Level 3 (BSL-3) facility at the University of Hong Kong. A/Hong Kong/VM24002346/2024 (A/HK/2346/2024) H9N2 viral isolate, from a specimen collected on day 1 post-symptom onset (pso), was provided by PHLC of the Centre for Health Protection (CHP) of Hong Kong.

A/Hong Kong/HKU-250128-1073/1999 (A/HK/1073/1999) H9N2 was isolated from a patient in Hong Kong in 1999 [2]. From the virus stock (P4), we have passaged the strains for 3 more passages before the virus replication experiments in organoids and MDCK cells.

### Live virus neutralization assay

Live virus neutralization assay was performed on MDCK cells. For statistical analysis, a value of 5 was assigned if the live virus-neutralizing antibody titre was <10.

### Establishment of human nasal and lung organoid

Human nasal and lung organoids were developed as described previously [22, 23]. Briefly, nasal cells were collected from the nasal mid-turbinate and lung cells were collected from normal lung tissue. The nasal and lung cells were embedded in 70% Matrigel (Corning^®^) in 24-well suspension culture plate and were expanded with expansion medium. To develop differentiated nasal and lung organoids, the undifferentiated 3D organoids were dissociated and seeded as 2D monolayer on Transwell^®^ (Corning^®^) plates and cultured in differentiation medium.

### Virus infection in MDCK cells and organoids

MDCK cells in 8-well chamber slides were inoculated with A/HK/1073/1999 or A/HK/2346/2024 at an MOI of 0.001, and were incubated at 37°C and 5% CO_2_ for 1 h. After incubation, the chambers were washed, replenished with fresh MEM with 2 µg/mL TPCK-trypsin, and incubated at 37°C in a 5% CO_2_ incubator. At 2, 24, 48 and 72 hpi, the cytopathic effect (CPE) was observed, and the supernatants were collected for viral titre determination. For immunofluorescence staining, the infected MDCK cells were fixed with 4% paraformaldehyde solution (PFA) and immunofluorescence staining was applied subsequently. All experiments were performed in triplicate.

2D human nasal and lung organoids were inoculated to the apical chamber with A/HK/1073/1999 and A/HK/2346/2024 at an MOI 0.001. After incubation at 37°C and 5% CO_2_ for 1 h, the inoculant was removed, and the apical chambers were washed and replenished with fresh basal medium. The infected organoids were incubated at 37°C in a 5% CO_2_ incubator. At each time point, the viral titre in the culture supernatant was determined by plaque assay. The Transwell inserts seeded with 2D organoids were fixed with 4% PFA, and immunofluorescence staining was performed. All experiments were performed in triplicate.

### Immunofluorescence staining for H9N2 NP

The 2D human organoids were inoculated with A/HK/1073/1999 or A/HK/2346/2024 at a multiplicity of infection (MOI) of 0.001. For negative control, basal medium was added instead of the H9N2 virus. Immunofluorescence staining for influenza virus was performed using the Influenza A DFA Screening Reagent from the D3 Ultra DFA Respiratory Virus Screening & ID Kit (Quidel).

### Immunofluorescence staining for 2,3 and 2,6-linked SA

Fixed nasal and lung organoids were incubated with biotinylated *Sambucus nigra* agglutinin (SNA) or biotinylated *Macckia amurensis* lectin II (MAL-II) (Vector Laboratories). Following washes with 0.1% PBST, the organoids were incubated with Streptavidin, AF647 Conjugate.

### Immunofluorescence staining for cilia

Fixed nasal and lung organoids were incubated with anti-beta-tubulin antibody. Following washes with 0.1% PBST, the organoids were incubated with Goat-anti-Mouse-IgG-AF555 (Invitrogen).

### Minigenome reporter assay

Luciferase activity-based minigenome reporter assay was performed as described previously [31]. Briefly, the PA, PB1, PB2 and NP derived from A/HK/1073/1999 or A/HK/2346/2024 in pcDNA3.1 vector were mixed with a luciferase reporter plasmid and a thymidine kinase promoter-*Renilla* luciferase reporter plasmid construct, and then co-transfected into HEK293 T cells and incubated at 37°C, 5% CO_2_ incubator. The luciferase activity was measured using a Dual-Luciferase Reporter Assay System (Promega) at 24 h post transfection. Polymerase activity was normalized against *Renilla* luciferase activity.

### Virus infection in mice

Male BALB/c mice (4–6 weeks old) were inoculated intranasally with 1 × 10^5^ PFU of A/HK/1073/1999 (*n* = 10) or A/HK/2346/2024 (*n* = 10). Five mice from each group were sacrificed on 4 dpi for viral titre determination in the nasal turbinate and lung, while another 5 mice from each group were monitored for body weight and survival for 10 days.

### Statistical analysis

Statistical analysis was performed using GraphPad Prism for Windows version 10.4.1. The viral titres were compared using multiple unpaired t test.

## Results

### Patient

The patient was a healthy 22-month-old girl (HK/2024 patient). She was hospitalized for influenza H9N2 infection in February 2024. The patient visited Zhongshan, China, from day-7 to day-3 before symptom onset. She did not have any direct poultry contact, consumption of undercooked poultry, or contact with patients with AIV infection. She first presented with fever and productive cough. Nasopharyngeal swab (NPS) collected on day 1 pso tested positive for influenza A virus by the multiplex PCR panel (BIOFIRE® Respiratory 2.1 plus Panel). On day 6 pso, the influenza A subtype was confirmed to be H9N2 by the PHLC, CHP of Hong Kong (A/Hong Kong/VM24002346/2024 [A/HK/2346/2024]).

On day 7 pso, she was admitted to Princess Margaret Hospital for isolation. Nasopharyngeal aspirate (NPA) collected on day 7 pso tested positive for influenza virus M, PB2 and H9 genes by the monoplex RT–PCR for M and H9 genes by an automated multiplex PCR panel (Avalon Automated Multiplex System). Other respiratory viruses were not detected using the two multiplex PCR panels (in-house panel at PHLC and Avalon Automated Multiplex System) and metagenomic RNA sequencing. Throat swab, eye swab and stool specimens tested negative for influenza virus with M and PB2 gene RT–PCR. Chest radiograph on day 7 pso did not show any pulmonary infiltrates. The patient was treated with oral oseltamivir 30 mg twice daily since day 7 pso for 5 days. The patient’s illness remained mild and did not require oxygen supplementation. Influenza virus RNA continued to be detected in the NPA collected on days 8 and 9 pso but was not detected in the NPA collected on day 10 pso.

H9N2 virus was successfully isolated with viral culture from the nasopharyngeal specimen collected on day 1 pso by the PHLC. Viral culture of subsequent nasopharyngeal specimens collected from day 7 to day 10 pso was unsuccessful.

### Household members

Out of the 3 household members living with the family, household member 1 had sore throat 2 days after the symptom onset of the index patient. The other 2 household members (household member 2 and 3) were asymptomatic. The combined nasopharyngeal and throat swab of all 3 household members tested negative for influenza virus by RT-PCR.

We also tested the paired serum specimens of the household members for the neutralization antibody (nAb) against the viral culture isolate from our patient (A/HK/2346/2024) and that of a human case strain in 1999 (A/HK/1073/1999) (Supplementary Table S4). The nAb titres against A/HK/1073/1999 were <10 for both acute and convalescent sera of all 3 household members. In contrast, for A/HK/2346/2024, household member 1 had a nAb titre of 10 for both acute and convalescent sera, while household members 2 and 3 both had nAb titre of <10 and 10 in the respective serum specimens.

### Viral replication in MDCK cells

We first compared the replication of A/HK/2346/2024 with A/HK/1073/1999 in MDCK cells. The CPE appeared earlier for A/HK/2346/2024 (first seen at 24 h post infection [hpi]) than that for A/HK/1073/1999 (first seen at 48 hpi) (Supplementary Figure S1A). Immunofluorescence staining showed a much higher proportion of cells being infected with A/HK/2346/2024 at 24 hpi when compared with A/HK/1073/1999 (Supplementary Figure S1B). All cells in the A/HK/2346/2024-infected chambers were detached at 72 hpi. The viral titres were statistically significantly higher for A/HK/2346/2024 than A/HK/1073/1999 at 24, 48 and 72 hpi (Supplementary Figure S1C). At 72 hpi, A/HK2346/2024 reached a titre of 8 log10 PFU/ml, while A/HK/1073/1999 only reached a titre of 5 log10 PFU/ml. Our results suggest that A/HK/2346/2024 replicated much faster and produces a higher yield than A/HK/1073/1999.

### Viral replication in human nasal and lung organoid

**Figure 1.**
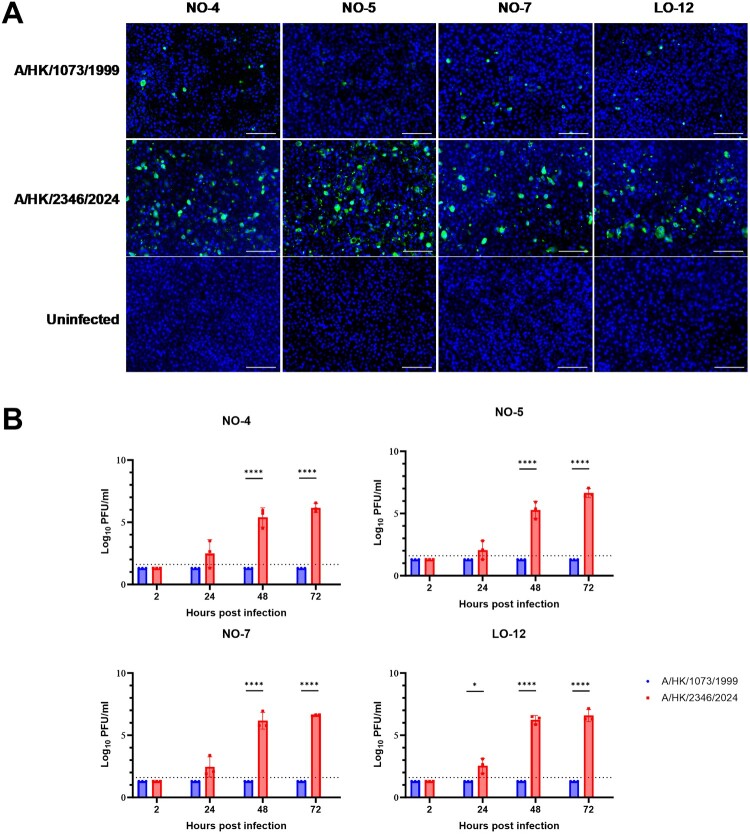
Comparison of 2024 and 1999 H9N2 viral infection in nasal and lung organoids. Differentiated nasal organoid from 3 donors (NO-4/NO-5/NO-7) and lung organoid from 1 donor (LO-12) were infected with A/HK/2346/2024 or A/HK/1073/1999 at an MOI of 0.001. For negative control (uninfected), basal medium was added instead of the H9N2 virus. (A) Immunofluorescence staining for AIV nucleoprotein: Immunofluorescence staining for AIV nucleoprotein at 72 hpi was performed. Scale bar, 100 μm. Magnification: 100× (B) Viral replication: The supernatants were collected from the apical chambers at 2, 24, 48 and 72 hpi for viral titre determination with plaque assay. Differences in viral titres between A/HK/1073/1999 or A/HK/2346/2024 were compared using the unpaired t test with log-transformed viral titres, with multiple comparisons corrected using the Benjamini, Krieger, and Yekutieli method. The experiment was performed in triplicate. Data represent mean ± standard deviation. Horizontal dotted lines indicate the lower limit of detection. A value of 20 (half of the lower detection limit) was assigned for samples with no PFU observed. *, *P* < .05; ****, *P* < .0001.

To determine whether the contemporary 2024 H9N2 isolate was better adapted to humans, we compared the replication of A/HK/2346/2024 with A/HK/1073/1999 in human nasal organoids from 3 donors and lung organoid from 1 donor. CPE was not observed for either virus at any time points. A/HK/2346/2024 replicated well in all 3 nasal organoids and the lung organoid. Immunofluorescence staining demonstrated that a higher proportion of cells in the nasal and lung organoids were infected with A/HK/2346/2024 when compared with A/HK/1073/1999 (Figure 1(A) and Supplementary Figure S2). Viral titres reached 6 log10 PFU in all organoids infected with A/HK/2346/2024 (Figure 1(B)). In contrast, viable virus could not be detected in the culture supernatant at any time points for A/HK/1073/1999 in any organoids. Taken together, these results suggest that the A/HK/2346/2024 is better adapted to both the upper and lower respiratory tract in humans than A/HK/1073/1999.

### Comparison of cellular tropism between A/HK/2346/2024 and A/HK/1073/1999

To determine if there are differences in cellular tropism between A/HK/2346/2024 and A/HK/1073/1999, we assessed the ratio of influenza NP-positive cells among ciliated and non-ciliated cells, MAL-II-positive (indicates the presence of 2,3-linked SA) or SNA-positive cells (indicate the presence of 2,6-linked SA) at 72 hpi (Table 1). In both nasal and lung organoids, A/HK/2346/2024 and A/HK/1073/1999 preferentially infected ciliated cells compared to non-ciliated cells. The ratio of infected 2,6-linked-SA/2,3-linked-SA cells were higher for A/HK/2346/2024 than A/HK/1073/1999 in both nasal organoid (1.53 vs 1.40) and lung organoid (2.93 vs 0.74). Therefore, the enhanced replication of A/HK/2346/2024 in human organoids may be attributed to a better infection of both ciliated and non-ciliated cells, and a stronger preference for 2,6-linked-SA.

**Table 1.** Differential cellular tropism of A/HK/2346/2024 and A/HK/1073/1999.

	% of infected cells				
Organoid	2,6-linked SA^c^	2,3-linked SA^d^	2,6/2,3 ratio	Ciliated cells	Non-ciliated cells
**Nasal**^a^					
A/HK/1073/1999	10.4	7.5	1.40	24.1	0.8
A/HK/2346/2024	34.3	22.4	1.53	56.8	5.9
					
**Lung**^b^					
A/HK/1073/1999	1.3	1.8	0.74	5.0	0.4
A/HK/2346/2024	23.4	8.0	2.93	59.8	3.5

Abbreviation: SA, sialic acid.

^a^ NO-5.

^b^ LO-12.

^c^ Cells positive for SNA.

^d^ Cells positive for MAL-II.

### Comparison of polymerase activity between A/HK/2346/2024 and A/HK/1073/1999

Viral polymerase activity is an important determinant of host adaptation. Here, we compared the viral polymerase activity between A/HK/2346/2024 and A/HK/1073/1999 using a minigenome reporter assay. We found that the viral polymerase activity was much weaker for A/HK/2346/2024 than that of A/HK/1073/1999 (Supplementary Figure S3). Hence, the enhanced replication of A/HK/2346/2024 is not related to differences in viral polymerase activity.

### Comparison of 1999 and 2024 H9N2 infection in mice

**Figure 2.**
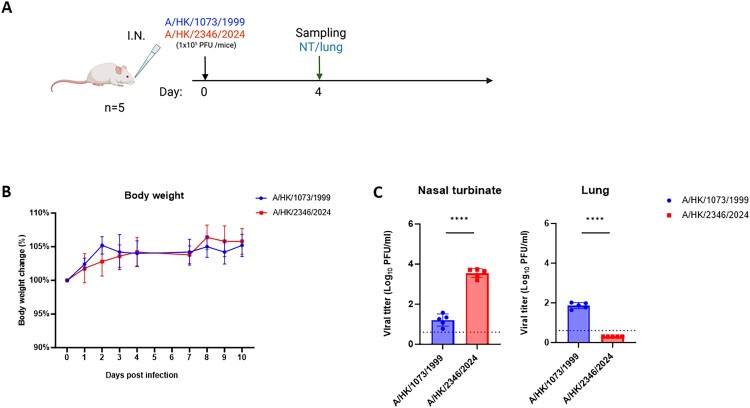
H9N2 infection in mice. (A) Schematic diagram of the experiment. (B) Body weight loss. Ordinary two-way ANOVA was used for statistical analysis. (C) Viral titres of nasal turbinate and lung were determined by plaque assay. Data represent mean ± standard deviation. Horizontal dotted line indicates the detection limit (4 PFU/ml). Unpaired t test was used for statistical analysis. ****, *P* < .0001.

The virulence and viral replication were assessed in a mouse model (Figure 2). All mice survived after infection with either A/HK/1073/1999 and A/HK/2346/2024, and there was no significant difference in body weight. On 4 dpi, mice infected with A/HK/2346/2024 had a significantly higher viral load in the nasal turbinate than those infected with A/HK/1073/1999. In contrast, mice infected with A/HK/1073/1999 had a significantly higher viral load in the lung than those infected with A/HK/2346/2024.

### Phylogenetic analysis

Nanopore sequencing was performed for the virus culture isolate from NPS on day 1 pso (Passage 1), and directly from NPA on days 7, 8, 9 and 10 pso. The coverage was sufficient for analysis only for the virus culture isolates from NPS collected on day 1 pso and the direct NPA specimen on day 7 pso. The nucleotide sequence of the virus culture isolate from day 1 pso specimen was 100% identical to that of the viral sequence obtained directly from the clinical specimen (EPI_ISL_18926219).

Phylogenetic analysis of the HA gene showed that the HK/2024 patient’s strain belonged to the Eurasian Y280/G9 h9.4.2.5 lineage group 2 [33, 34] (Figure 3(A)). The HA, NA and PA genes were most closely related to recent human H9N2 cases. The HA gene was most closely related to the human H9N2 cases in Guangdong in 2021 (A/Guangdong/00470/2021) and 2020 (A/Guangdong/20SF15010/2020) (Figure 3(A) and Supplementary Table S5); the NA gene was most closely related to a 2020 human H9N2 case in Guangdong (A/Guangdong/SF16348/2020) (Figure 3(B) and Supplementary Table S6); while the PA gene was most closely related to a human H9N2 case from Sichuan, China in 2023 (A/Yann/001/2023) (Figure 3(C)). The PB2 gene was most closely related to an H3N8 strain isolated from patients in 2023 (A/Guangdong/ZS-23SF005/2023) and 2022 (A/Changsha/1000/2022), and to an H9N2 strain from a patient in 2021 (A/Guangdong/21ASF101/2021) (Figure 3(D)). Both PA and PB2 genes were also closely related to chicken H3N8 from Guangdong in 2021. Figure 3.Phylogenetic trees showing the relationship between the nucleotide sequences of our patient and other human or avian influenza H9N2 strains. (A) HA; (B) NA; (C) PA; (D) PB2. The phylogenetic trees were constructed using maximum likelihood method with best fit model of nucleotide substitution using IQTree v3.0.1 ([A] GTR+F+G4; [B] GTR+F+G4 [C] GTR+F+G4; [D] GTR+F+G4). The phylogenetic trees were visualized and exported using FigTree v1.4.4. The bootstrap values from 1000 replicates were performed to evaluate the reliability of phylogenetic trees. Sequences not determined in this study were obtained from the Global Initiative on Sharing All Influenza Data (GISAID) EpiFlu^TM^ Database (Supplementary Table S8).

**Figure:**
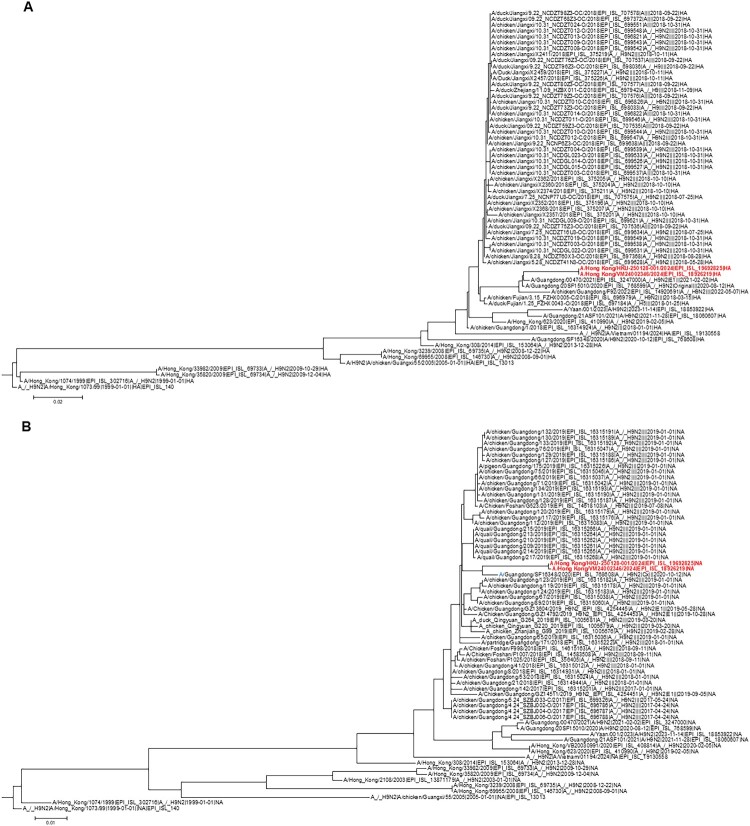

**Figure:**
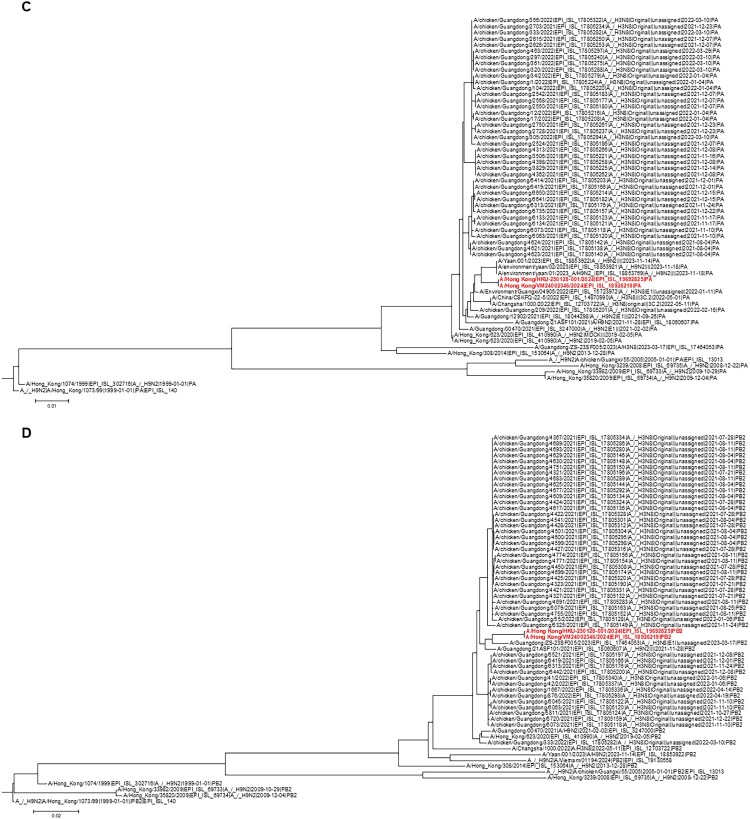

The PB1, NP, NS and M genes were closely related to those of H3N8 or H9N2 in poultry from China, but only distantly related to human strains. The PB1 gene was more closely related to H3N8 than other H9N2 viruses (Supplementary Figure S4A). The NS gene was closely related to chicken H3N8 and environmental H9N2 viruses (Supplementary Figure S4B). The NP gene was most closely related to H9N2 poultry isolates (Supplementary Figure S4C). However, the M gene is most closely related to poultry H9N2 from China (Supplementary Figure S4D).

### Analysis of amino acid substitutions associated with mammalian adaptation

Our patient’s strain carries several HA aa substitutions that are associated with either enhanced binding to α2,6-linked-SA or enhanced airborne transmission [35], including 183N, 190 T, 192R and 226L (H3 numbering [36]) (Supplementary Table S7). The NA contains a 3-aa deletion at residues 62–64 (N2 numbering) which is associated with enhanced entry into human cells [20]. The PB2 contains the A588 V substitution which has been associated with enhanced polymerase activity in human cells and enhanced virulence in mice [37], and K702R which is over-represented in human influenza viruses H1N1 and H3N2 [38]. The PB1 contains 368 V, one of the residues that is found in an H5N1 virus that is airborne transmissible in ferrets [39]. The PA contains K356R which is over-represented among the H7N9 that causes human outbreaks [40], and S409N which is over-represented among human H9N2 [41]. The M1 protein contains the R95 K, which has been associated with the escape from TRIM21 host restriction and more severe disease in mice [42]. Analysis of the NA and PA proteins did not show any aa substitutions associated with resistance to NA inhibitors or baloxavir, respectively. All these aa substitutions, except HA 226L, were not present in A/HK/1073/1999.

### *De novo* substitutions emerging during infection

When compared with the aa sequence of the specimen collected on day 1 pso, *de novo* nucleotide substitutions (>50%) have emerged at 3 sites for the nasopharyngeal specimen collected on day 7 pso (A/Hong Kong/HKU-250128-001/2024; GISAID accession number: EPI_ISL_19692825). These include the synonymous C483 T substitution in the NA gene (52%; coverage 114×), the non-synonymous G757A (D253N) substitution in the PB2 gene (66%; coverage 56×), and the non-synonymous C55 T (R19C; coverage 23×) substitution in the body domain of NP gene (52%). All these mutations were not detected in the specimen collected on day 1 pso, even as minor variant.

The PB2 D253N substitution has been associated with enhanced polymerase activity in mammalian cells [43, 44]. The NP protein may play a role in host restriction by evading the host restriction factor MxA. Hence, we determined whether the NP-R19C residue is located at the binding site to MxA. However, molecular docking study suggests that the NP residue 19 does not participate in the binding between NP and MxA (Supplementary Figure S5). Both PB2 D253N and NP R19C substitutions are rarely found in human or avian H9N2 strains (Table 1).

### Comparison of mammalian adaptive substitutions between human and avian H9N2 strains

**Figure 4.**
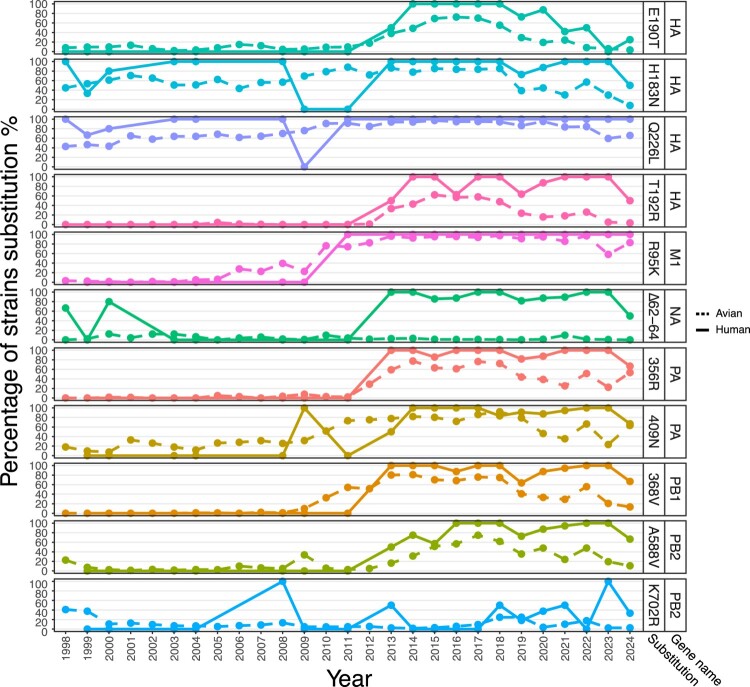
Global temporal trends in strain substitution percentages for H9N2 gene segments. The annual percentages of strains carrying specific mammalian adaptive aa substitutions in avian (dotted lines) and human (solid lines) H9N2 viruses are shown. Y-axis indicates the percentage of strains harbouring the respective substitutions, while x-axis represents the year of collection. Each aa substitution within a specific gene segment is shown as a uniquely coloured line, with both the substitution and its gene segment labelled on the right, highlighting temporal trends in the acquisition and persistence of these mutations, with several adaptive substitutions becoming more prevalent over time in human isolates.

Next, we determined the prevalence of mammalian adaptive substitutions among human and avian H9N2 strains from 1998 to 2024. The proportion of mammalian adaptive substitutions has generally increased over the years, and is generally higher among human than avian H9N2 strains, especially for HA T192R, NA Δ62-64, PB1 I368V and PB2 A588V (Table 2 and Figure 4).

**Table 2.** Markers of mammalian adaptation present in avian-origin and human-origin influenza A H9N2 viruses.

		Frequency of substitutions in influenza A H9N2 viruses, no/total (%)	
Protein^a^	Amino acid substitution	Human	Avian
HA^b^	H183N	79/91 (86.81%)	9,957/13,422 (74.18%)
	E190T	52/91 (57.14%)	5,655/13,421 (42.14%)
	T192R	62/91 (68.13%)	4,580/13,421 (34.13%)
	Q226L	87/91 (95.6%)	11,812/13,421 (88.01%)
NA	Δ62-64	71/93 (76.34%)	166/7,032 (2.36%)
PB2	D253N^c^	0/83 (0%)	6/5,997 (0.1%)
	A588V	62/83 (74.7%)	2,277/5,997 (37.97%)
	K702R	21/83 (25.3%)	685/5,995 (11.43%)
PB1	I368V	65/83 (78.31%)	3,149/5,939 (53.02%)
PA	K356R	68/83 (81.93%)	2,834/5,877 (48.22%)
	S409N	69/83 (83.13%)	3,976/5,877 (67.65%)
NP	R19C^c^	0/83 (0%)	1/6,064 (0.02%)
M1	R95K	75/83 (90.36%)	4,846/6,246 (77.59%)

^a^ Based on references 22, 35, 37, 38, 39, 40, 41.

^b^ H3 numbering system was used.

^c^ Only found in the nasopharyngeal specimen collected on day 7 PSO.

## Discussion

H9N2 is the second most common AIV subtype that causes human infections since 2018. We aimed to evaluate the zoonotic potential of contemporary H9N2 virus strains in humans. We observed that an H9N2 virus strain obtained from a patient in 2024 replicated much better in human nasal and lung organoids compared to an H9N2 virus strain isolated from a patient infected in 1999. Furthermore, we identified several human adaptive mutations in the 2024 H9N2 strain, including the PB2-D253N mutation which was rarely found in H9N2 strains from humans and only emerged on day 7 pso of our patient. Our results suggest a potential increase in adaptation of contemporary H9N2 strains in humans.

Viral load in the upper respiratory tract correlates with person-to-person transmissibility [45]. In this study, we showed that the H9N2 strain from HK/2024 patient replicated better in the human nasal organoids than an H9N2 strain isolated from a patient in 1999, suggesting the possibility that the contemporary strain may be more likely to cause person-to-person transmission. We have specifically chosen human nasal organoid model because these consist of ciliated cells, goblet cells, club cells and basal cells [21] and therefore physiologically similar to the human upper respiratory tract. The human nasal organoid model complements the studies in mammalian models for respiratory droplet transmission, such as those in ferrets and guinea pigs [46].

The 2024 H9N2 virus also replicated well in lung organoids. H9N2 has been documented to cause severe lower respiratory tract infections, especially among immunocompromised patients [5]. However, in the mice experiment, viable virus could not be detected in the lung of 2024 H9N2-infected mice on 4 dpi. Hence, infection in mice model has failed to recapitulate the replication in human airway.

Our human organoid model, which recapitulate the cell types present in human tissues, is well-suited to assess the difference in cellular tropism between the 2024 and 1999 H9N2 strains. We demonstrated that both strains preferentially infected ciliated cells than non-ciliated cells, which corroborates with the findings of Matrosovich *et al*. [32]. Furthermore, we found that the 2024 H9N2 strain has a higher predilection to infect 2,6-linked-SA than 2,3-linked-SA. Further studies are required to determine the molecular mechanisms underlying the differences in cellular tropism.

H9N2 is the donor of internal genes for AIVs that cause severe human infections. Hence, the identification of mutations in H9N2 internal proteins that facilitate human adaptation is crucial for assessing the zoonotic risk of not only H9N2, but also other AIV subtypes. Unlike previous studies which only compared single viral genomes derived from single specimens of each patient, we also assessed the *de novo* substitutions that emerge during infection. Our strategy avoids missing adaptive mutations that facilitate human infection but emerge later during infection. In our patient, we have identified the emergence of *de novo* mutations at two amino residues, PB2-D253N and NP-R19C, on day 7 pso. Notably, PB2-D253N, has been previously demonstrated to affect polymerase activity in mammalian cells [43, 44]. Mok *et al* showed that an H9N2 virus with PB2 D253N substitution plays a synergistic role with Q591K in enhancing the polymerase activity, TNF-α production in human macrophages, replication in human bronchial epithelial cells, and pathogenic in mice [43]. Zhang *et al*. showed that PB2-D253N mutation has enhanced polymerase activity in human 293T cells [44].

The nasopharyngeal viral load of our patient decreased rapidly since day 7 pso, which is likely a result of the initiation of oseltamivir and the immune clearance and therefore the virus cannot further adapt or transmit to others. However, if an immunocompromised patient is infected with H9N2, the virus can persist for a long time that allows the accumulation of human adaptive mutations. This is well demonstrated for SARS-CoV-2, for which the virus can persist for a long period among immunocompromised patients, and the emergence of adaptive mutations that favour replication [47]. Therefore, it is important to monitor for these mutations that only emerge in humans.

The phylogenetic analysis showed that HA, NA, PA and PB2 genes were closely related to H9N2 or H3N8 from humans. These results suggest that HA, NA, PA and PB2 genes may have already gained fitness advantage for human adaptation. For HA, it contains the 183N, 190T, 192R and 226L mutations which have been associated with increased binding to 2,6 SLN or increase airborne transmission [48]. The NA contains the 3-aa stalk deletion which is characteristic of enhanced entry into human cells [20].

The internal genes PA, PB2, PB1, NS and NP of A/HK/2346/2024 is closely related to both H9N2 and H3N8. There is a possibility of reassortment between H9N2 and H3N8. Further mixing of gene pools between H9N2 and H3N8 may give rise to future human strains.

There are several limitations in this study. First, since we failed to isolate the virus from the nasopharyngeal specimen collected on day 7 pso, we could not compare the viral fitness between the virus with the *de novo* mutations. Second, we compared only one contemporary strain with a historical strain. Additional comparisons with other human strains will be required to validate the generalizability of our findings.

H9N2 has been relatively neglected as a potential pandemic agent. Over the years, H9N2 has already acquired numerous mutations that facilitate adaptation to humans [35]. In this study, we demonstrated the enhanced fitness of the contemporary H9N2 isolate in human nasal organoid and identified the emergence of human adaptive mutations that only appeared late during infection. Being a reservoir of internal genes for AIVs that cause severe human infections, H9N2 can significantly contribute to an AIV that causes a human pandemic. Having a low pathogenicity in birds, H9N2 can spread widely in wild birds without being noticed. In fact, H9N2 is the predominant AIV subtype detected among poultry [19]. Antigenic drift has allowed the virus to evade from vaccine-induced immunity [49]. With widespread co-circulation of H5N1 2.3.3.4b and H9N2 in wild birds [50], there is an increased chance of reassortment between these two strains. If an H5N1 2.3.4.4b, which already circulates widely in mammals, reassorts with an H9 virus, there is a high risk of an endemic or even a pandemic virus. Continual genotypic and phenotypic surveillance of H9N2, especially from human and mammalian infections, plays a pivotal role in the assessment of zoonotic risk of all AIVs [51].

## Supplementary Material

Supplemental Material

Supplemental Material

6_Supplementary_Table_S8_20250908.xls
