# Supplementary material for: Enhanced replication of a contemporary avian influenza A H9N2 virus in human respiratory organoids
Source: Emerg Microbes Infect. 2025 Oct 16;14(1):2576574. doi: 10.1080/22221751.2025.2576574 (PMC12584838; doi:10.1080/22221751.2025.2576574)
Supplement: Supplemental Material [file TEMI_A_2576574_SM9806.pdf]

SUPPLEMENTARY MATERIALS

Supplementary Table S1. Primers and probes used in this study.

Primer / probe	Sequence (5'to 3')	Gene target	Reference
FLUAM-7-F	CTTCTAACCGAGGTCGAAACGTA	Matrix	(1)
FLUAM-161-R	GGTGACAGGATTGGTCTTGTCTTTA		
FLUAM-49-P6	FAM- TCAGGCCCCCTCAAAGCCGAG -BHQ1		
FluA_PB2-F	GACGTRGTGTTGGTAATGAAAC	PB2	(2)
FluA_PB2-R	GAATYCTTTTGGTCGCTGTCTG		
FluA_PB2-Probe	FAM- AAACGGGACTCTAGYATACTTACTGACAG -IABkFQ		
H9_Inner-Fm	GTYAAGCTGGAATCTGARGG	HA	Modified from (1)
H9_Inner-Rm	AAGGCAGCAAACCCCAT		
H9_Pbm	HEX- CTCACYATTTATTCGACTGTCGCCTCATCT -lABkFQ		

Supplementary Table S2. Pathogen targets of the respiratory infectious disease assay panel.

Pathogen target	PHLC panel	AAMST panel
Adenovirus	√	√
Bocavirus		√
Coronavirus 229E	√	√
Coronavirus NL63		√
Coronavirus HKU1		√
Coronavirus OC43	√	√
SARS-CoV-2	√	√
MERS-CoV		√
Enterovirus/Rhinovirus	√	√
Influenza A	√	√
Influenza A (pdm09 H1)	√	√
Influenza A (H2)		√
Influenza A (H3)	√	√
Influenza A (H5)		√
Influenza A (H6)		√
Influenza A (H7)		√
Influenza A (H9)		√
Influenza A (H10)		√
Influenza B	√	√
Influenza C	√	√
Metapneumovirus	√	√
Parainfluenza 1	√	√
Parainfluenza 2	√	√
Parainfluenza 3	√	√
Parainfluenza 4	√	√
Parechovirus		√
RSV	√	√

Abbreviations: AAMST, Avalon Automated Multiplex System; MERS-CoV, Middle East respiratory syndrome coronavirus; PHLC, Public Health Laboratory Centre; RSV, respiratory syncytial virus; SARS-CoV-2, severe acute respiratory syndrome coronavirus 2

Supplementary Table S3. Accession number of H9N2 sequences.

Specimen	GISAID name	GISAID accession number
NPS (Day 1 pso)	A/Hong Kong/VM24002346/2024	EPI_ISL_18926219
NPA (Day 7 pso)	A/Hong Kong/HKU-250128-001/2024	EPI_ISL_19692825
A/HK/1073/1999 culture isolate[a]	A/Hong Kong/HKU-250128-1073/1999[c]	EPI_ISL_19692826
A/HK/2346/2024 culture isolate[b]	A/Hong Kong/HKU-250128-2346/2024	EPI_ISL_19692824

Abbreviations: NPA, nasopharyngeal aspirate; NPS, nasopharyngeal swab

[a] Cultured in Madin Darby canine kidney cells

[b] Cultured from nasopharyngeal specimen collected on day 1 pso

[c] Sequence obtained for the stock that was used in the replication experiment

Supplementary Table S4. Neutralization antibody titers of acute and convalescent sera of the 3 household members.

Virus	Household member	Neutralization antibody titer	
		**Acute[b]**	**Convalescent[c]**
**A/HK/1073/1999**	**1[c]**	<10	<10
	**2**	<10	<10
	**3**	<10	<10
**A/HK/2346/2024**	**1[d]**	10	10
	**2**	<10	10
	**3**	<10	10

[a] The limit of detection is 10.
[b] The serum specimens were collected on day 9 or day 10 after the symptom onset of the index patient
[c] The serum specimens were collected on day 32 after the symptom onset of the index patient
[d] Household member 1 reported sore throat 2 days after the symptom onset of the index patient.

Supplementary Table S5. Amino acid differences in the hemagglutinin between HK/2024 patient and the most closely related human cases in Figure 2A.

Protein	Amino acid residue (H9 numbering[a])	Amino acid residue (H3 numbering[a])	HK/2024 patient		Guangdong patients	
			Day 1 pso	Day 7 pso	A/Guangdong/20SF15010/2020	A/Guangdong/00470/2021
HA	7		M	M	I	I
	104	94	A	A	V	V
	112	101	S	S	N	N
	122	111	V	V	F	F
	127	116	K	K	R	R
	145	133	N	N	D	D
	195	187	S	S	T	T
	196	188	E	E	D	D
	217	209	M	M	I	I
	232	224	S	S	N	N
	264	NA	H	H	Y	Y
	283	274	K	K	R	R

[a] The amino acid numbering follows the system by Burke et al. (3)

Exclude signal peptide

Supplementary Table S6. Amino acid differences in the neuraminidase protein between HK/2024 patient and the most closely related human case.

Amino acid residue[a]	HK/2024 patient		Guangdong patients
	Day 1 pso	Day 7 pso	A/Guangdong/SF16348/2020
33	M	M	L
43	R	R	S
46	S	S	L
124	D	D	Y
191	I	I	V
301	S	S	Y
307	H	H	Y

[a] N2 numbering

Supplementary Table S7. Mammalian adaptive substitutions from published literature.

**Protein**		**Substitutions**[a]	**Virus strain**	
	Wild type		**Day 1 pso**	**Day 7 pso**
**PB2**	D	**D253N**	D	N
	T	T271A	T	T
	I	**I292V**	V	V
	K	K340R	R	R
	K	K526R	K	K
	A	A558V	E	E
	A	**A588V**	V	V
	G	G590S	G	G
	Q	Q591K	Q	Q
	E	E627K/V	E	E
	D	D701N	D	D
	K	**K702R**	R	R
**PB1**	H	H99Y	H	H
	D	D120T	D	D
	S	S261Y	S	S
	R	R327K	R	R
	V	V336I	V	V
	I	**I368V**	V	V
	D	D439Y	D	D
**PA**	K	**K26E**	E	E
	T	T85I	T	T
	M	M86S	M	M
	V	V100A	I	I
	L	L336M	L	L
	K	K356R	R	R
	S	S409N	N	N
**HA (H3 numbering)**	I	I155T	Y	Y
	D	**D183N**	N	N
	D	**D190T**/V	T	T
	Q	**Q192R**	R	R
	Q	**Q226L**	L	L
	S	S227Q	M	M
	G	G228S	G	G
**NA (N2 numbering)**		Δ62-64	Δ	Δ
**M1**	R	**R95K**	K	K
**M2**	S	**S31N**/G	N	N

Abbreviations: pso, post symptom onset

[a] Based on references (4-7)

Supplementary Figure S1. Comparison of 2024 and 1999 H9N2 viral infection in Madin Darby canine kidney (MDCK) cells. MDCK cells were infected with A/HK/2346/2024 or A/HK/1073/1999 at an MOI of 0.001. For negative control, MEM medium was added instead of the H9N2 virus.

(A) **Cytopathic effects**: Images of MDCK cells were taken at 2, 24, 48 and 72 hpi. Red arrows indicate influenza-induced cytopathic effects. Magnification: 40×

(B) **Immunofluorescence staining for influenza A nucleoprotein**: Immunofluorescence staining for influenza A nucleoprotein was performed using Influenza A DFA Screening Reagent from the D3 Ultra DFA Respiratory Virus Screening & ID Kit. Scale bar = 100 μm. Magnification: 100×

(C) **Viral replication**: The supernatants were collected from the apical chambers at 2, 24, 48 and 72 hpi for the determination of viral titers with plaque assay. Data represented mean ± SD. Horizontal dotted line indicates the detection limit (40 PFU/ml). The difference in viral titers between A/HK/1073/1999 or A/HK/2346/2024 were compared using the unpaired t test with log-transformed viral loads, with multiple comparisons corrected using the Benjamini, Krieger, and Yekutieli method. The experiment was performed in triplicate. ****, $P<0.0001$

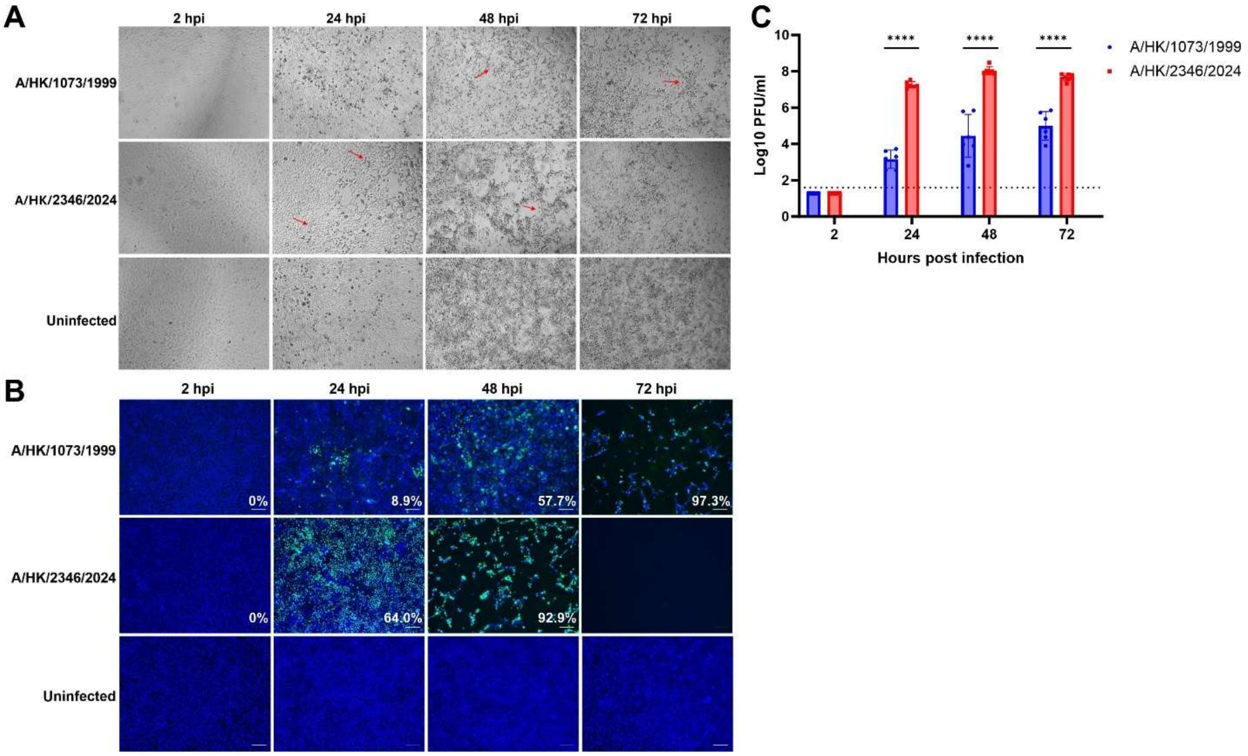

Supplementary Figure S2. Immunofluorescence staining for influenza A nucleoprotein in human nasal and lung organoids. Differentiated human nasal organoid monolayers from 3 donors (NO-4, NO-5, NO-7) and human lung organoid monolayer from 1 donor (LO-12) were infected with A/HK/2346/2024 or A/HK/1073/1999 at an MOI of 0.001. Immunofluorescence staining for influenza A nucleoprotein was performed using Influenza A DFA Screening Reagent from the D3 Ultra DFA Respiratory Virus Screening & ID Kit. Scale bar = 100 μm. Magnification: 100×

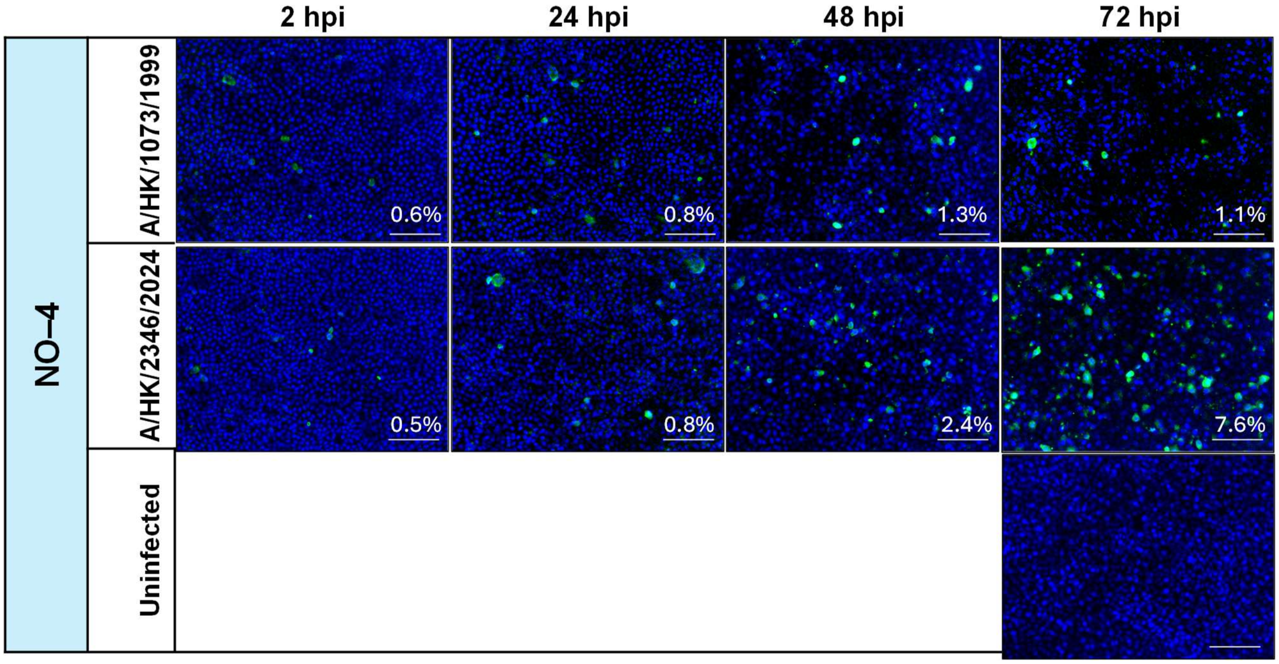

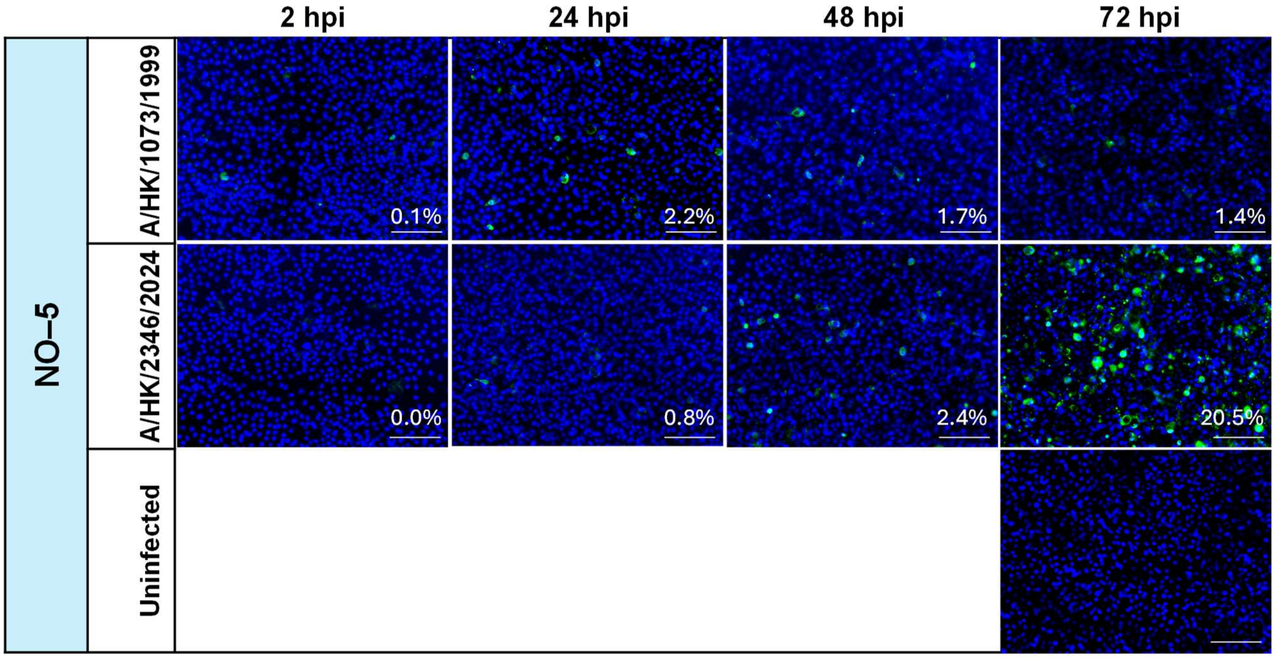

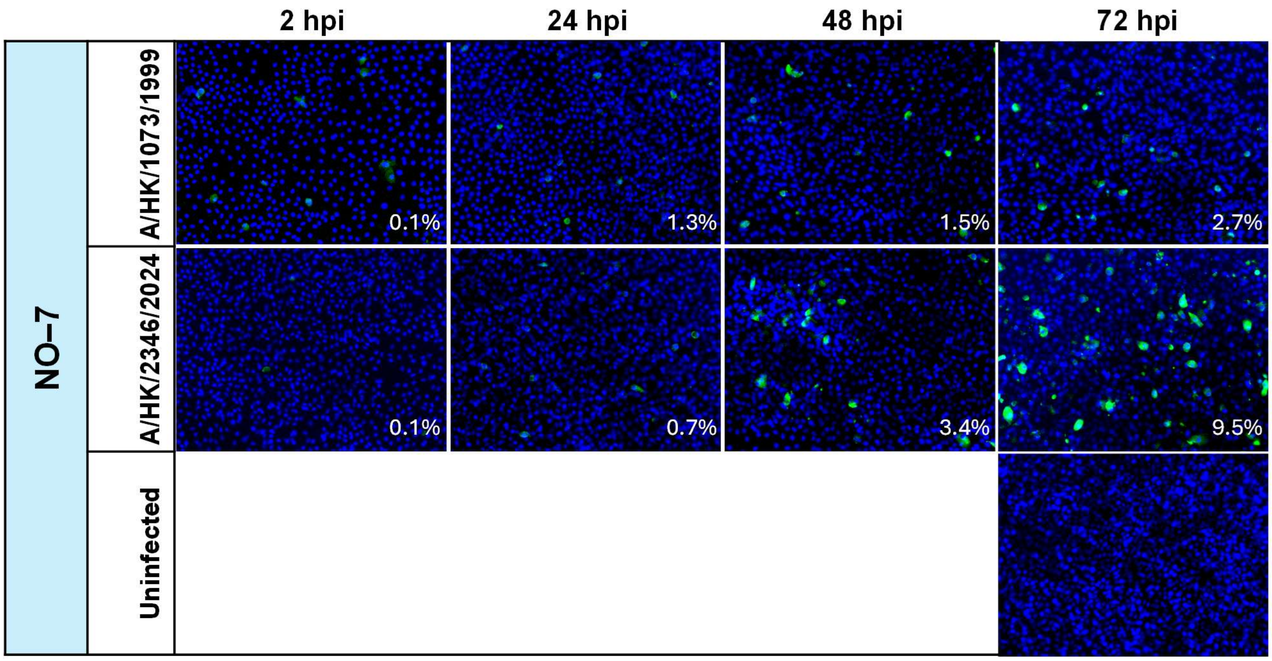
2 hpi
24 hpi
48 hpi
72 hpi
NO–7
A/HK/1073/1999
A/HK/2346/2024
Uninfected
0.1%
1.3%
1.5%
2.7%
0.1%
0.7%
3.4%
9.5%

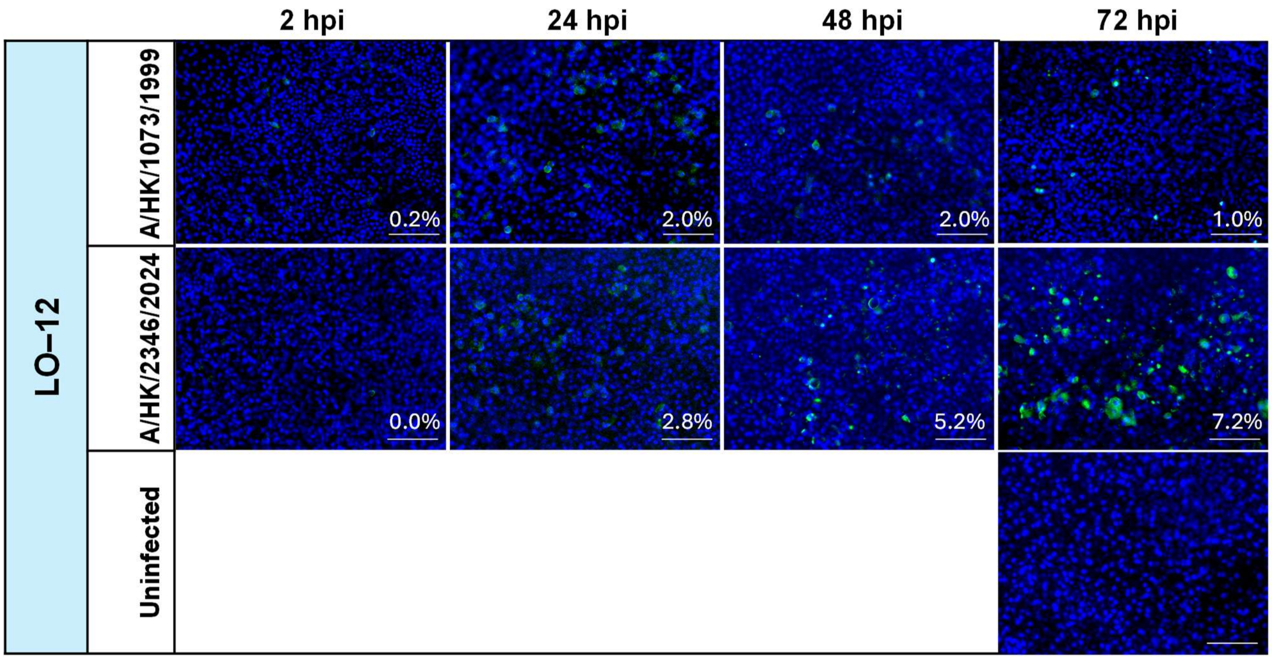
2 hpi
24 hpi
48 hpi
72 hpi
LO–12
A/HK/1073/1999
A/HK/2346/2024
Uninfected
0.2%
2.0%
2.0%
1.0%
0.0%
2.8%
5.2%
7.2%

Supplementary Figure S3. Comparison of polymerase activity between A/HK/2346/2024 and A/HK/1073/1999. HEK293T cells were transfected with H9N2 RNP complexes composed of NP, PB1, PB2 and PA from A/HK/2346/2024 or A/HK/1073/1999, together with firefly luciferase reporter and a Renilla luciferase reporter. Luciferase activity was measured at 24 h post transfection. The RNA polymerase activity was normalized against the *Renilla* luciferase activity. Data represent mean ± SD of the relative luciferase activity (luciferase activity normalized against Renilla luciferase activity) from four independent experiments. RNP without the PB2 gene from each virus represented as blank control. Statistical significance was analysed by one-way analysis of variance, corrected by the Bonferroni post-test: **** $P<0.0001$.

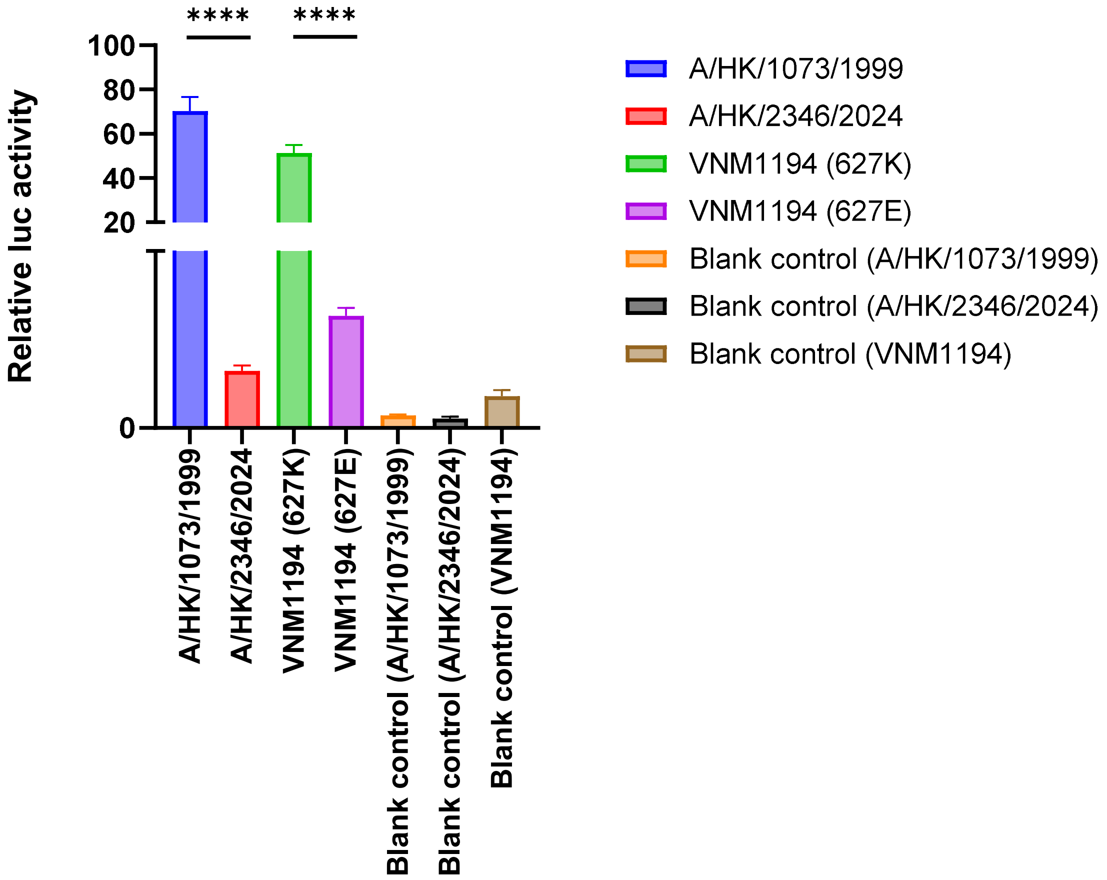

Supplementary Figure S4. Phylogenetic trees showing the relationship between the nucleotide sequences of HK/2024 patient and other human or avian influenza H9N2 strains. (A) PB1; (B) NS; (C) NP; (D) M. The phylogenetic trees were constructed using maximum likelihood method with best fit model of nucleotide substitution using IQTree2 v2.3.1 [A] GTR+F+G4; [B] TIM2+F+I; [C] K3Pu+F+G4; [D] TVMe+G4). The phylogenetic trees were visualized and exported using FigTree v1.4.4. The bootstrap values from 1000 replicates were performed to evaluate the reliability of phylogenetic trees. Sequences not determined in this study were obtained from the Global Initiative on Sharing All Influenza Data (GISAID) EpiFlu^{TM} Database (Supplementary Table S8).

A

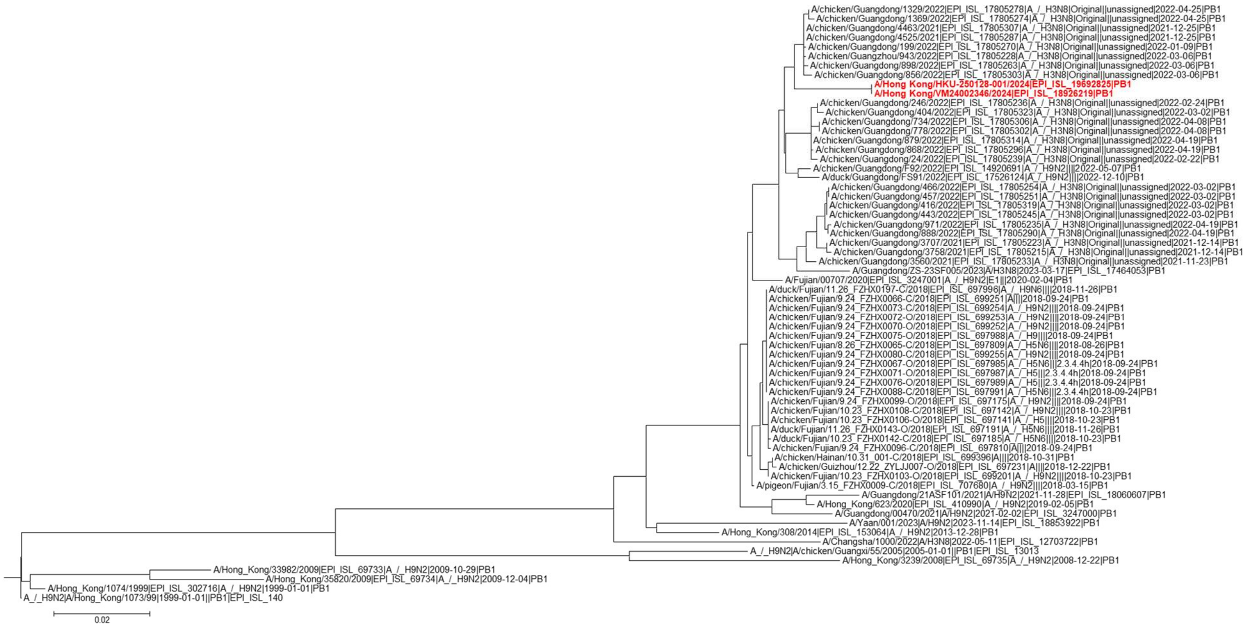

B

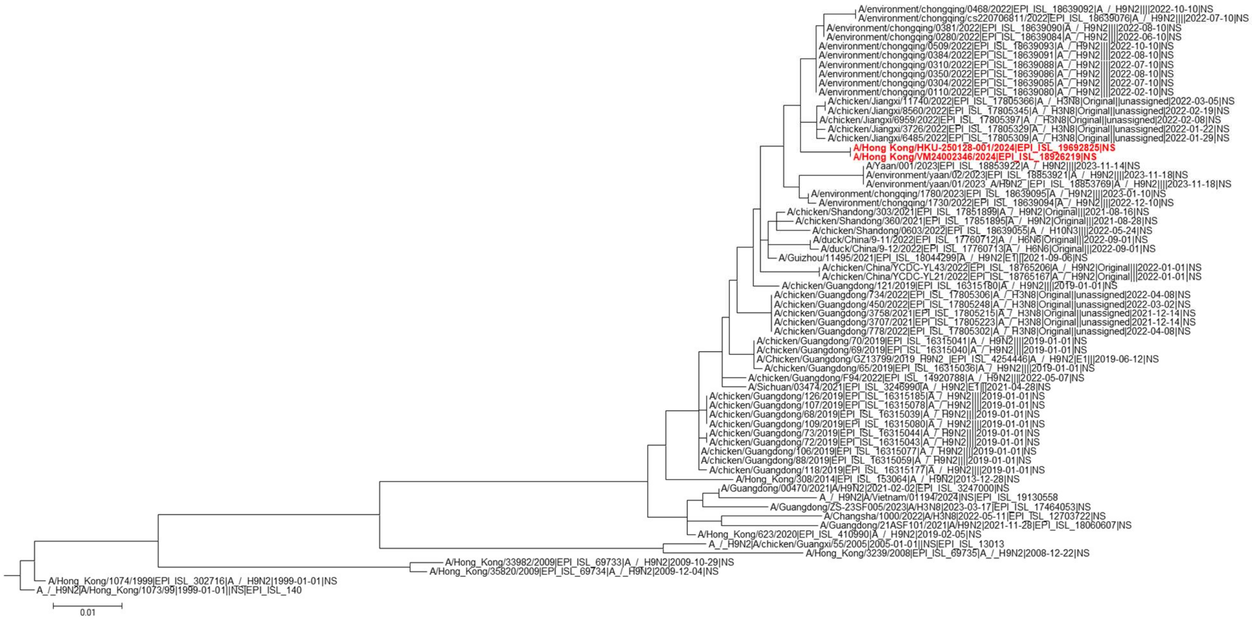

C

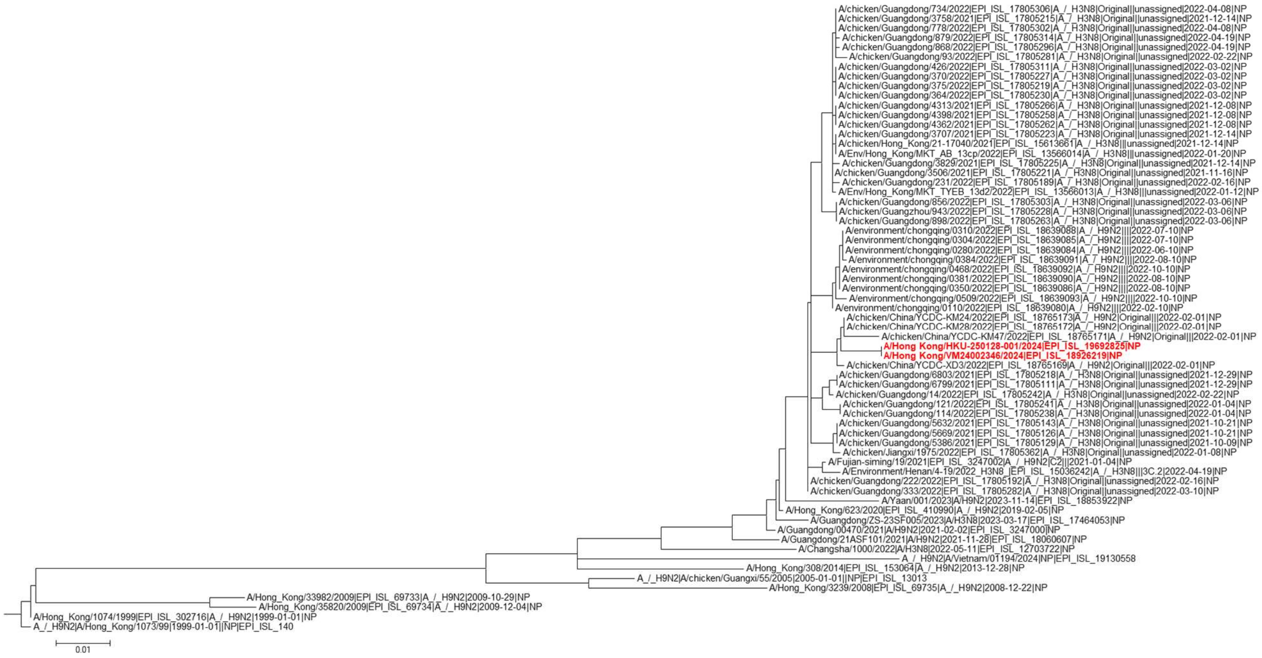

D

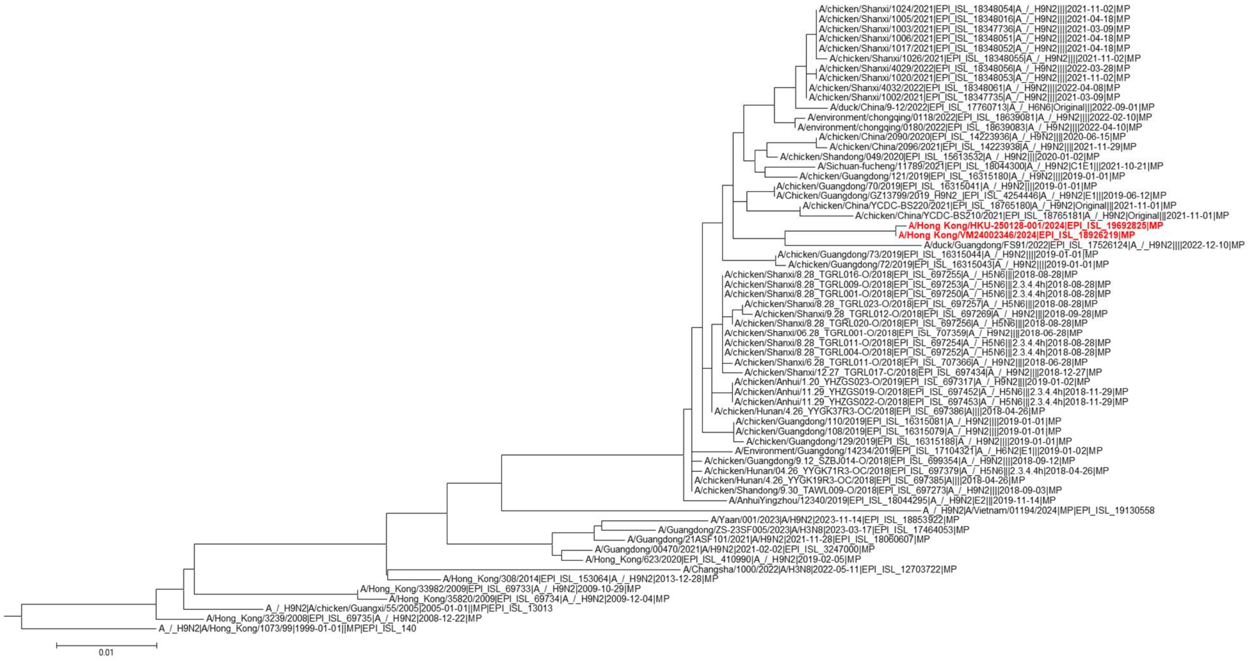

A/chicken/Shanxi/1024/2021|EPI_ISL_18348054|A_/_H9N2||||2021-11-02|MP
A/chicken/Shanxi/1005/2021|EPI_ISL_18348016|A_/_H9N2||||2021-04-18|MP
A/chicken/Shanxi/1003/2021|EPI_ISL_18347736|A_/_H9N2||||2021-03-09|MP
A/chicken/Shanxi/1006/2021|EPI_ISL_18348051|A_/_H9N2||||2021-04-18|MP
A/chicken/Shanxi/1017/2021|EPI_ISL_18348052|A_/_H9N2||||2021-04-18|MP
A/chicken/Shanxi/1026/2021|EPI_ISL_18348055|A_/_H9N2||||2021-11-02|MP
A/chicken/Shanxi/4029/2022|EPI_ISL_18348056|A_/_H9N2||||2022-03-28|MP
A/chicken/Shanxi/1020/2021|EPI_ISL_18348053|A_/_H9N2||||2021-11-02|MP
A/chicken/Shanxi/4032/2022|EPI_ISL_18348061|A_/_H9N2||||2022-04-08|MP
A/chicken/Shanxi/1002/2021|EPI_ISL_18347735|A_/_H9N2||||2021-03-09|MP
A/duck/China/9-12/2022|EPI_ISL_17760713|A_/_H6N6|Original|||2022-09-01|MP
A/environment/chongqing/0118/2022|EPI_ISL_18639081|A_/_H9N2||||2022-02-10|MP
A/environment/chongqing/0180/2022|EPI_ISL_18639083|A_/_H9N2||||2022-04-10|MP
A/chicken/China/2090/2020|EPI_ISL_14223936|A_/_H9N2||||2020-06-15|MP
A/chicken/China/2096/2021|EPI_ISL_14223938|A_/_H9N2||||2021-11-29|MP
A/chicken/Shandong/049/2020|EPI_ISL_15613532|A_/_H9N2||||2020-01-02|MP
A/Sichuan-fucheng/11789/2021|EPI_ISL_18044300|A_/_H9N2|C1E1|||2021-10-21|MP
A/chicken/Guangdong/121/2019|EPI_ISL_16315180|A_/_H9N2||||2019-01-01|MP
A/chicken/Guangdong/70/2019|EPI_ISL_16315041|A_/_H9N2||||2019-01-01|MP
A/Chicken/Guangdong/GZ13799/2019_H9N2_|EPI_ISL_4254446|A_/_H9N2|E1|||2019-06-12|MP
A/chicken/China/YCDC-BS220/2021|EPI_ISL_18765180|A_/_H9N2|Original|||2021-11-01|MP
A/chicken/China/YCDC-BS210/2021|EPI_ISL_18765181|A_/_H9N2|Original|||2021-11-01|MP
A/Hong Kong/HKU-250128-001/2024|EPI_ISL_19692825|MP
A/Hong Kong/VM24002346/2024|EPI_ISL_18926219|MP
A/duck/Guangdong/FS91/2022|EPI_ISL_17526124|A_/_H9N2||||2022-12-10|MP
A/chicken/Guangdong/73/2019|EPI_ISL_16315044|A_/_H9N2||||2019-01-01|MP
A/chicken/Guangdong/72/2019|EPI_ISL_16315043|A_/_H9N2||||2019-01-01|MP
A/chicken/Shanxi/8.28_TGRL016-O/2018|EPI_ISL_697255|A_/_H5N6||||2018-08-28|MP
A/chicken/Shanxi/8.28_TGRL009-O/2018|EPI_ISL_697253|A_/_H5N6|||2.3.4.4h|2018-08-28|MP
A/chicken/Shanxi/8.28_TGRL001-O/2018|EPI_ISL_697250|A_/_H5N6|||2.3.4.4h|2018-08-28|MP
A/chicken/Shanxi/8.28_TGRL023-O/2018|EPI_ISL_697257|A_/_H5N6||||2018-08-28|MP
A/chicken/Shanxi/9.28_TGRL012-O/2018|EPI_ISL_697269|A_/_H9N2||||2018-09-28|MP
A/chicken/Shanxi/8.28_TGRL020-O/2018|EPI_ISL_697256|A_/_H5N6||||2018-08-28|MP
A/chicken/Shanxi/06.28_TGRL001-O/2018|EPI_ISL_707359|A_/_H9N2||||2018-06-28|MP
A/chicken/Shanxi/8.28_TGRL011-O/2018|EPI_ISL_697254|A_/_H5N6|||2.3.4.4h|2018-08-28|MP
A/chicken/Shanxi/8.28_TGRL004-O/2018|EPI_ISL_697252|A_/_H5N6|||2.3.4.4h|2018-08-28|MP
A/chicken/Shanxi/6.28_TGRL011-O/2018|EPI_ISL_707366|A_/_H9N2||||2018-06-28|MP
A/chicken/Shanxi/12.27_TGRL017-C/2018|EPI_ISL_697434|A_/_H9N2||||2018-12-27|MP
A/chicken/Anhui/1.20_YHZGS023-O/2019|EPI_ISL_697317|A_/_H9N2||||2019-01-02|MP
A/chicken/Anhui/11.29_YHZGS019-O/2018|EPI_ISL_697452|A_/_H5N6|||2.3.4.4h|2018-11-29|MP
A/chicken/Anhui/11.29_YHZGS022-O/2018|EPI_ISL_697453|A_/_H5N6|||2.3.4.4h|2018-11-29|MP
A/chicken/Hunan/4.26_YYGK37R3-OC/2018|EPI_ISL_697386|A||||2018-04-26|MP
A/chicken/Guangdong/110/2019|EPI_ISL_16315081|A_/_H9N2||||2019-01-01|MP
A/chicken/Guangdong/108/2019|EPI_ISL_16315079|A_/_H9N2||||2019-01-01|MP
A/chicken/Guangdong/129/2019|EPI_ISL_16315188|A_/_H9N2||||2019-01-01|MP
A/Environment/Guangdong/14234/2019|EPI_ISL_17104321|A_/_H6N2|E1|||2019-01-02|MP
A/chicken/Guangdong/9.12_SZBJ014-O/2018|EPI_ISL_699354|A_/_H9N2||||2018-09-12|MP
A/chicken/Hunan/04.26_YYGK71R3-OC/2018|EPI_ISL_697379|A_/_H5N6|||2.3.4.4h|2018-04-26|MP
A/chicken/Hunan/4.26_YYGK19R3-OC/2018|EPI_ISL_697385|A||||2018-04-26|MP
A/chicken/Shandong/9.30_TAWL009-O/2018|EPI_ISL_697273|A_/_H9N2||||2018-09-03|MP
A/AnhuiYingzhou/12340/2019|EPI_ISL_18044295|A_/_H9N2|E2|||2019-11-14|MP
A_/_H9N2|A/Vietnam/01194/2024|MP|EPI_ISL_19130558
A/Yaan/001/2023|A/H9N2|2023-11-14|EPI_ISL_18853922|MP
A/Guangdong/ZS-23SF005/2023|A/H3N8|2023-03-17|EPI_ISL_17464053|MP
A/Guangdong/21ASF101/2021|A/H9N2|2021-11-28|EPI_ISL_18060607|MP
A/Guangdong/00470/2021|A/H9N2|2021-02-02|EPI_ISL_3247000|MP
A/Hong_Kong/623/2020|EPI_ISL_410990|A_/_H9N2|2019-02-05|MP
A/Changsha/1000/2022|A/H3N8|2022-05-11|EPI_ISL_12703722|MP
A/Hong_Kong/308/2014|EPI_ISL_153064|A_/_H9N2|2013-12-28|MP
A/Hong_Kong/33982/2009|EPI_ISL_69733|A_/_H9N2|2009-10-29|MP
A/Hong_Kong/35820/2009|EPI_ISL_69734|A_/_H9N2|2009-12-04|MP
A_/_H9N2|A/chicken/Guangxi/55/2005|2005-01-01||MP|EPI_ISL_13013
A/Hong_Kong/3239/2008|EPI_ISL_69735|A_/_H9N2|2008-12-22|MP
A_/_H9N2|A/Hong_Kong/1073/99|1999-01-01||MP|EPI_ISL_140
0.01

Supplementary Figure S5. Effect of NP R19C on the binding between NP and human MxA protein.

(A) Experimentally identified MX1-resistant NP substitutions/sites, adopted from (8). Amino acid sites of NP that exhibit only minor contribution to MX1 resistance were highlighted in light red, whereas amino acid sites that strongly increased MX1 resistance were indicated in red. 1918 refers to the 1918 pandemic H1N1, while pH1N1 refers to the 2009 H1N1pdm09.

(B) Experimentally identified potential MX1-resistant substitutions/sites and site 19 were highlighted on AlphaFold2-predicted H9N2 NP structure in red and green, respectively.

(C) Predicted MX1 dimer-H9N2 NP interacting pose by using Rosetta protein-protein docking protocol. MX1 dimer structure was modeled with AlphaFold2-multimer. The MX1 dimer substrate-binding domain was constrained to be close to those amino acid sites that strongly increase MX1 resistance as indicated in red. As demonstrated, site 19 is distant from the interacting interface. Therefore, substitution R19C was predicted to have no role in MX1 resistance.

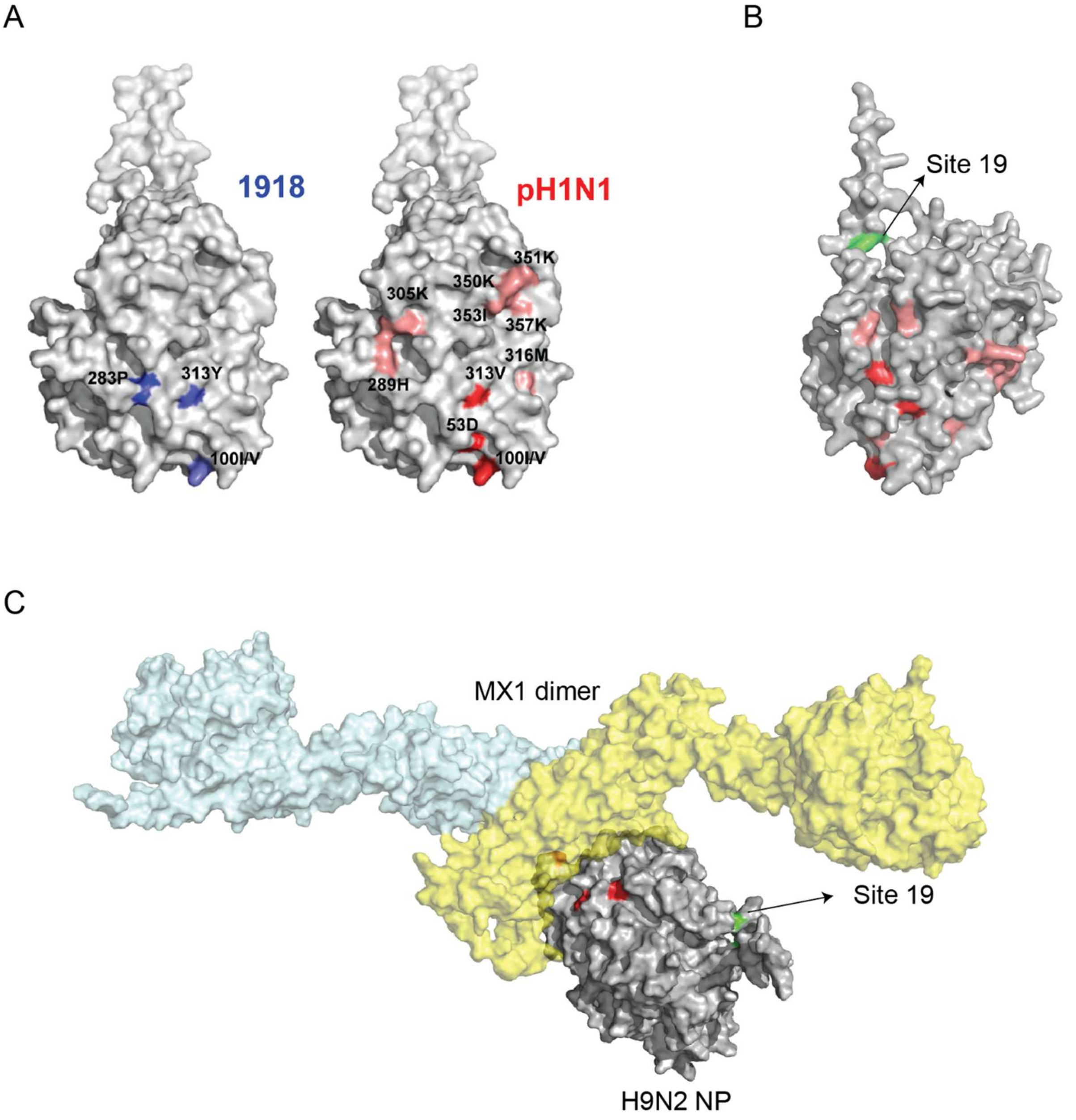
A
1918
pH1N1
283P
313Y
100I/V
305K
350K
351K
353I
357K
316M
289H
313V
53D
100I/V
B
Site 19
C
MX1 dimer
Site 19
H9N2 NP

REFERENCES

1. World Health Organization. WHO information for the molecular detection of influenza viruses. Revision February 2021. Available at https://cdn.who.int/media/docs/default-source/influenza/molecular-detention-of-influenza-viruses/protocols_influenza_virus_detection_feb_2021.pdf?sfvrsn=df7d268a_5. Accessed on 11th April 2024 2021.
2. Yip CC, Chan WM, Ip JD, Seng CW, Leung KH, Poon RW, et al. Nanopore Sequencing Reveals Novel Targets for Detection and Surveillance of Human and Avian Influenza A Viruses. J Clin Microbiol. 2020;58(5).
3. Burke DF, Smith DJ. A recommended numbering scheme for influenza A HA subtypes. PLoS One. 2014;9(11):e112302.
4. Arai Y, Elgendy EM, Daidoji T, Ibrahim MS, Ono T, Sriwilaijaroen N, et al. H9N2 Influenza Virus Infections in Human Cells Require a Balance between Neuraminidase Sialidase Activity and Hemagglutinin Receptor Affinity. J Virol. 2020;94(18).
5. Tan M, Zeng X, Xie Y, Li X, Liu J, Yang J, et al. Reported human infections of H9N2 avian influenza virus in China in 2021. Front Public Health. 2023;11:1255969.
6. Herfst S, Schrauwen EJ, Linster M, Chutinimitkul S, de Wit E, Munster VJ, et al. Airborne transmission of influenza A/H5N1 virus between ferrets. Science. 2012;336(6088):1534-41.
7. Imai M, Watanabe T, Hatta M, Das SC, Ozawa M, Shinya K, et al. Experimental adaptation of an influenza H5 HA confers respiratory droplet transmission to a reassortant H5 HA/H1N1 virus in ferrets. Nature. 2012;486(7403):420-8.
8. Manz B, Dornfeld D, Gotz V, Zell R, Zimmermann P, Haller O, et al. Pandemic influenza A viruses escape from restriction by human MxA through adaptive mutations in the nucleoprotein. PLoS Pathog. 2013;9(3):e1003279.
