# Supplementary material for: Enhanced replication of a contemporary avian influenza A H9N2 virus in human respiratory organoids
Source: Emerg Microbes Infect. 2025 Oct 16;14(1):2576574. doi: 10.1080/22221751.2025.2576574 (PMC12584838; doi:10.1080/22221751.2025.2576574)
Supplement: Supplemental Material [file TEMI_A_2576574_SM9681.pdf]

Supplementary Methods

Influenza virus RT-PCR

One-step real-time RT-PCR assays for influenza virus detection were performed using QuantiNova Probe RT-PCR Kit (QIAGEN). The primers and probes used in this study were shown in Supplementary Table S1 [1, 2]. The reagent mixture (20 μL) contained 10 μL of 2× QuantiNova Probe RT-PCR Master Mix, 0.2 μL of QN Probe RT-Mix, 1.6 μL of each 10 μM forward and reverse primer, 0.4 μL of 10 μM probe, 1.2 μL of nuclease-free water and 5 μL of TNA as the template. The thermocycling condition was 10 min at 45 °C for reverse transcription and 5 min at 95 °C for PCR initial activation, followed by 45 cycles of 5 s at 95 °C and 30 s at 55 °C. All reactions were performed using the LightCycler 96 Instrument (Roche, Basel, Switzerland).

Whole genome sequencing and bioinformatics analysis for influenza virus

Influenza virus H9N2 whole genome sequencing was performed using the Oxford Nanopore MinION device (Oxford Nanopore Technologies, Oxford, United Kingdom). Total nucleic acid was amplified with SuperScript™ III One-Step RT-PCR System with Platinum™ Taq High Fidelity DNA Polymerase (Thermo Fisher Scientific, Waltham, MA, USA) using ONT-Uni12 (5'- TTTCTGTTGGTGCTGATATTGCAGCAAAAGCAGG -3'), ONT-Uni12G (5'- TTTCTGTTGGTGCTGATATTGCAGCGAAAGCAGG-3') and ONT-Uni13 (5'- ACTTGCCTGTCGCTCTATCTTCAGTAGAAACAAGG -3'). RT-PCR conditions were 1 cycle of 42 °C for 15 min, 55°C for 15 min, 60°C for 5 min, 94 °C for 2 min; 5 cycles of 94 °C for 30 s, 45 °C for 30 s and 68 °C for 3 min; followed by 30 cycles of 94 °C for 30 s, 57 °C for 30 s and 68 °C for 3 min. PCR products were purified with 1× AMPure XP beads (Beckman Coulter, Brea, CA, USA) and quantified using Qubit dsDNA HS Assay Kit (Thermo Fisher Scientific, Waltham, Massachusetts, United States). The purified PCR products were then

normalized and added with Rapid Barcodes using Rapid Barcoding Kit (SQK-RBK114.96, Oxford Nanopore Technologies, Oxford, United Kingdom). Barcoded libraries were then pooled, purified with 1× AMPure XP beads and then quantified using Qubit dsDNA HS Assay Kit. Purified pooled libraries were then added with sequencing adapters and sequenced with the Oxford Nanopore MinION device using R10.4.1 flow cells for 48 hours.

The consensus sequence was generated by mapping the reads to the H9N2 genome EPI_ISL_18926219 using Medaka v1.11.3 (https://github.com/nanoporetech/medaka). The read alignment results were visualized using Integrative Genomics Viewer (IGV) v2.17.3 (10.1038/nbt.1754). The minor allele frequencies (MAF) at the reported locations were also determined using the IGV. The consensus sequences were deposited into GISAID (Supplementary Table S7).

Phylogenetic analysis

All segments of the consensus sequences were compared against the Influenza Nucleotide Database of GISAID using MEGABLAST with default parameters. The 50 strains with the highest score were selected for phylogenetic analysis. We have also included all previous human H9N2 from Hong Kong. For all trees, H9N2 A/chicken/GuangXi/55/2005 was chosen as the representative strain. Eurasian Y280/G9 h9.4.2.5, and A/Hong Kong/1073/99 was chosen as the outgroup to structure the tree properly. The gene segments of the resulting strains were then downloaded from GISAID. The gene segments were aligned with A/Hong Kong/1073/99(H9N2) using MAFFT v7.520 [3].

The aligned nucleotide sequences were used to construct phylogenetics trees. The phylogenetic trees were constructed using maximum likelihood method using IQTree2 v2.3.1 [4]. The bootstrap values from 1000 replicates were performed to evaluate the reliability of

phylogenetic trees. The phylogenetic trees were visualized and exported using FigTree v1.4.4 (https://github.com/rambaut/figtree).

Metagenomic sequencing

Metagenomic sequencing was performed using the Oxford Nanopore MinION device (Oxford Nanopore Technologies, Oxford, United Kingdom). Library preparation using the sequence-independent single-primer amplification (SISPA) method was described previously [5]. Total nucleic acid was first treated with DNase (TURBO DNA-free Kit, Invitrogen, Thermo Fisher Scientific, Waltham, MA, USA) to remove residual DNA. The DNase-treated RNA was concentrated using RNA Clean & Concentrator-5 Zymo-Spin IC Column (Zymo Research, Irvine, CA, USA). Concentrated RNA was reverse transcribed with SuperScript™ IV Reverse Transcriptase (Thermo Fisher Scientific, Waltham, MA, USA) to single strand complementary DNA (cDNA) using primer A (5'-GTTTCCCACTGGAGGATA-N9-3') followed by second strand cDNA synthesis using Klenow Fragment (3'→5' exo-) (New England BioLabs, Ipswich, MA, USA). cDNA was then amplified by PCR using primer B (5'-GTTTCCCACTGGAGGATA-3'). Nanopore sequencing library preparation was performed according to manufacturer's instructions for Native Barcoding Kit 96 V14 (SQK-NBD114.24, Oxford Nanopore Technologies, Oxford, United Kingdom). Briefly, amplified PCR products were purified by 1× AMPure XP bead (Beckman Coulter, California, CA). An equal molar of each amplified PCR product was then subjected to DNA repair, end preparation, and native barcode ligation. Barcoded libraries were pooled, ligated to sequencing adaptor and sequenced with the Oxford Nanopore MinION device using R10.4.1 flow cell for 48 hours.

Frequency of human adaptation substitutions in H9N2 sequences

We downloaded all amino acid sequences of human and avian H9N2 from GISAID on 11th February 2025, following a uniform naming format: "Protein Accession no. | Gene name | Isolate name | Isolate ID | Type | Collection date". Since some sequences retrieved may be duplicated or originate from the same isolate, we manually curated a representative set of isolates in Excel, prioritizing those with the most complete segments data. Then, these selected sequences were aligned using MAFFT v7.526 [6].

We further identified and quantified the amino acid mutation for each targeted position using an in-house R script. The script parsed multi-aligned protein sequences with R package seqinr v4.2.36 and assigned residue numbering based on a user-defined reference sequence. We selected A/H3N2|A/Aichi/2/1968|HA|EPI_ISL_123225 as the H3 numbering reference for HA segments. For NA, NP, PA, PB1, PB2, and M1 segments, mutations were identified based on their respective segments from A/Hong_Kong/1073/99|EPI_ISL_140. To ensure accurate positioning, gaps (represented by "-" in sequences) in the reference sequence were ignored when assigning mutation positions. The script identified mutations by analyzing each sequence individually. For single amino acid substitutions, it extracted the amino acid at the target position based on the reference-mapped numbering. A mutation was counted if this amino acid matched the specified mutated residue. For deletion mutations, the script checked all positions within the specified target area. A deletion was counted only if every position in this area contains an alignment gap. Since each isolate may have multiple sequences per gene segment, we only retained the longest sequence per gene per isolate when multiple sequences were available to prevent redundancy in mutation count calculations.

Mutation frequency on a yearly basis was calculated as the number of isolates carrying the mutation divided by the total number of non-redundant sequences collected that year. To visualize annual trends, these frequencies were plotted using the ggplot2 v3.5.1 package, with collection dates grouped into yearly intervals via R package lubridate v1.9.4. This selection

was performed using the R package dplyr v1.1.4 for grouping and filtering. Finally, the cleaned dataset, including protein sequences, isolate metadata, and mutation counts, was exported for further analysis, ensuring reproducibility and accuracy in mutation frequency estimation. To validate the results, we manually cross-checked mutation count statistics using Unipro UGENE [7], confirming the accuracy of the automated mutation calling analysis.

Comparison of amino acid sequences

The nucleotide sequences of different gene segments of A/Guangdong/20SF15010/2020, A/Guangdong/00470/2021, Day 1 PSO, and Day 7 PSO were translated into amino acid sequences separately using BioEdit v7.2.5 [8]. The amino acid sequences from the same gene segment were then aligned using MAFFT v7.520 [3] and then visualized using BioEdit. Amino acid mutations in any of the Day 1 PSO and Day 7 PSO strains were identified if they were not found in A/Guangdong/20SF15010/2020 or A/Guangdong/00470/2021.

Protein-protein docking between H9N2 nucleoprotein (NP) and MX1

Full-length structures of mutant (R19C) H9N2 NP and human MX1 dimer were predicted with AlphaFold2 [9] and AlphaFold2-multimer [10], respectively. Crystal structure of MX1 stalk (PDB id: 3LJB) was used as a template for AlphaFold2-multimer prediction. The predicted structures were relaxed with Amber [11]. NP surface patch containing experimentally identified potential MX1-resistant NP sites (53, 100, 289, 305, 313, 316, 350, 351, 353, 357) [12] was docked against the MX1 L4 loop dimer region which was known to be critical in influenza A virus NP binding. Protein-protein docking simulation was performed by using Rosetta high-resolution full-atom docking protocol [13], only small random perturbations (3 Å translation and 8° rotation) were applied to the docking partners. The number of output

structures was set to 10000 and the top 20 output structures (ranked by interface score) were manually analyzed with PyMol.

Viral culture from clinical specimens

Viral culture was performed on MDCK cells (ATCC CCL-34) in the Biosafety Level 3 facility at the University of Hong Kong. A/HK/2346/2024 H9N2 viral isolate, from a specimen collected on day 1 PSO, was kindly provided by PHLC of the Centre for Health Protection of Hong Kong. A/HK/1073/1999 H9N2 was isolated from a patient in Hong Kong in 1999 [14]. Briefly, 50 µL of viral isolates were inoculated to a T25 flask of MDCK cells with 1mL minimum essential medium (MEM) (Gibco, Thermo Fisher Scientific, Waltham, MA, USA). After incubation at 37°C and 5% CO_2 for 1 h, the inoculant was removed and replenished with 5 mL of MEM containing 2 µg/mL trypsin treated with L-1-tosylamido-2-phenylethyl chloromethyl ketone (TPCK) (Sigma-Aldrich, Life Science, USA), 100 U/mL penicillin and 100 µg/mL streptomycin, and incubated at 37°C and 5% CO_2 for 3 days. Viral cultures expressing >50% CPE were harvested. All virus stocks were stored at -80°C before use.

For A/HK/1073/1999, we retrieved the stock which was previously passaged in MDCK cells. From this virus stock (P4), we have performed 3 additional passages in MDCK cells before the virus infection experiments in organoids and MDCK cells. For A/HK/2346/2024 H9N2, the virus stock P3 was used for the infection experiments.

Live virus neutralization assay

Live virus nAb assay was performed on MDCK cells in the Biosafety Level 3 facility at the University of Hong Kong. Briefly, serum samples were heat-inactivated at 56 °C for 30 min and were serially diluted in 2-folds with MEM. Duplicates of each diluted serum were mixed with 100 50% tissue culture infectious dose ($TCID_{50}$) of A/HK/2346/2024 (GISAID

accession number: EPI_ISL_19692824) or A/HK/1073/1999 virus (GISAID accession number: EPI_ISL_19692826) at 37°C and 5% CO_2 for one hour. After incubation, 100 μL of the serum-virus mixture was then added to MDCK that were seeded in 96-well plates 24 h before infection. After incubation at 37°C and 5% CO_2 for one hour, the serum-virus mixture was removed and replenished with 100 μL of MEM containing 2 μg/mL TPCK trypsin, 100 U/mL penicillin and 100 μg/mL streptomycin, and incubated at 37°C and 5% CO_2 for 3 days. The live virus NAb titer was determined as the highest dilution with 50% inhibition of cytopathic effects. For statistical analysis, a value of 5 was assigned if the live virus-neutralizing antibody titer is <10.

Establishment of human nasal and lung organoid

Human nasal and lung organoids were developed as described previously [15, 16]. Briefly, nasal cells collected from the nasal mid-turbinate and lung cells collected from normal lung tissue were processed and embedded in 70% Matrigel (Corning®) in a 24-well suspension culture plate (Thermo Scientific™). The organoids were supplemented with expansion medium and passaged every 7 to 14 days.

To develop differentiated nasal and lung organoid monolayers, undifferentiated 3D organoids were digested into single cells with 10X TrypLE™ Select for 3-5 minutes at 37°C. Resultant singles cells were seeded onto Transwell inserts (Corning®). The cells were cultured in expansion medium to reach 90% confluency. Differentiation was then initiated by switching to differentiation medium (PneumaCult™-ALI Medium [STEMCELL Technologies]) and incubated for another 10 to 12 days. The medium was supplied to both the apical and basolateral chambers of the Transwell and was replenished every other day.

Virus infection in MDCK cells

MDCK cells in 8-well chamber slides (Cat no: 154534; Thermo Scientific, Waltham, MA, USA) were inoculated with A/HK/1073/1999 or A/HK/2346/2024 at an MOI of 0.001, and were incubated at 37°C and 5% CO_2 for 1 h. For negative control, MEM medium was added instead of the H9N2 virus. After the incubation, the inoculant was removed, and the chambers were washed with MEM, and were replenished with fresh MEM with 2 μg/mL TPCK-trypsin. The cells were incubated at 37 °C in a 5% CO2 incubator. The CPE of MDCK cells were observed at 2, 24, 48 and 72 hpi to evaluate the replications of the two H9 viruses. The supernatants were collected at 2, 24, 48 and 72 hpi to detect the viral titers. The infected MDCK cells in 8-well chamber slides were fixed with 4% paraformaldehyde solution (PFA) at 2, 24, 48 and 72 hpi, and immune fluorescence staining was applied subsequently.

Virus infection in human nasal and lung organoids

2D human nasal and lung organoids were inoculated with A/HK/1073/1999 and A/HK/2346/2024 at an MOI 0.001 by adding the virus inoculum to the apical chamber. For negative control, basal medium was added instead of the H9N2 virus. After incubation at 37°C and 5% CO_2 for 1 h, the inoculant was removed, and the apical chambers were washed and replenished with fresh basal medium. The infected organoid cells were incubated at 37 °C in a 5% CO_2 incubator. The supernatants were collected from the apical chambers at 2, 24, 48 and 72 hpi. At each of the time points, the viral titer in the culture supernatant was determined by plaque assay. The Transwell inserts seeded with 2D organoids were fixed with 4% paraformaldehyde solution (PFA), and immune fluorescence staining was applied subsequently.

Determination of viral titer by plaque assay

The MDCK cells were seeded one day before infection at 1×10^5 cells/well in 24-well plates. On the day of infection, cells were infected with 10-fold serial dilutions of culture

supernatant and incubated at 37°C, 5% CO_2 incubator for 1 hour. After incubation, the inoculum was removed and replaced with 1× MEM medium containing 2 μg/mL TPCK trypsin and 1% low-melting point agarose solution. Three days after infection, the cells were fixed with 4% formaldehyde in phosphate buffered saline (PBS) overnight and stained with 0.5% crystal violet solution to reveal the plaques. Virus titers were calculated as PFU/mL.

Immunofluorescence staining

The 2D human nasal and lung organoids were inoculated with A/HK/1073/1999 or A/HK/2346/2024 at a MOI of 0.001. For negative control, basal medium was added instead of the H9N2 virus. Immunofluorescence staining was performed using the Influenza A DFA Screening Reagent from the D3 Ultra DFA Respiratory Virus Screening & ID Kit (Quidel). Briefly, the organoids were fixed with 4% paraformaldehyde. After washing with 1X PBS for 3 times, the organoids were incubated with the reagent on an orbital shaker overnight at 4 °C. After overnight incubation, the organoids were washed with 1 x PBS for 3 times, and mounted with ProLong^{TM} Gold Antifade Mountant. The images were acquired using an Olympus IX73 microscope.

Immunofluorescence staining for cilia

Fixed nasal and lung organoids were permeabilized with 0.1% Triton X-100 in PBS, blocked with a 2% BSA and 2% goat serum in PBS, and incubated with anti-beta-tubulin antibody (Sigma). Following three washes with 0.1% PBST, the organoids were incubated with Goat-anti-Mouse-IgG-AF555 (Invitrogen). After final washes with 0.1% PBST, the organoids were mounted with VECTASHIELD® Antifade Mounting Medium with DAPI. Images were acquired using an Olympus BX53F microscope.

Quantitative analysis

For each stained organoid, a single 100x image was acquired at the central of the Transwell. Total cell counts were determined from thresholded DAPI channel images using the Analyze Particles function in ImageJ (National Institute of Health). The number of cells positive for NP, α-2,3 sialic acid, α-2,6 sialic acid, and β-tubulin were quantified by manual counting.

Minigenome reporter assay

Luciferase activity-based minigenome reporter assay was performed as described previously (31). Polymerase complexes composed of PA, PB1, PB2 and NP derived from A/HK/1073/1999 or A/HK/2346/2024 and cloned into pcDNA3.1 vector (50 ng each) were mixed with a luciferase reporter plasmid (50 ng) and a thymidine kinase promoter-*Renilla* luciferase reporter plasmid (pRL_TK) construct (10 ng), then co-transfected into HEK293T cells by Lipofectamine 3000 transfection kit (Invitrogen) and incubated at 37°C, 5% CO_2 incubator. RNP complex components derived from H5N1 (A/VNM/1194/04) together with its PB2 627K or PB2 627E were used as the positive and negative controls for the experiment. The luciferase activity was measured using a Dual-Luciferase Reporter Assay System (Promega) at 24 h post transfection. RNP polymerase activity was normalized against *Renilla* luciferase activity.

Virus infection in mice

Male BALB/c mice (4–6 weeks old) were obtained from the Centre for Comparative Medicine Research of the University of Hong Kong. The experimental procedures were approved by the Animal Ethics Committee on the Use of Live Animals in Teaching and Research of HKU (CULATR 24-281). Briefly, mice were randomly grouped and inoculated

intranasally with 1 × 10^5 PFU of A/HK/1073/1999 (n=10) or A/HK/2346/2024 (n=10) in 20 µl volume per mice. Five mice from each group were sacrificed on day 4 for viral titer determination in the nasal turbinate and lung, while another 5 mice from each group was monitored for body weight and survival for a total of 10 days. The nasal turbinate and lungs were lysed by magnetic beads in 1 ml Dulbecco's Modified Eagle Medium (DMEM) for the determination of viral titer using plaque assay.

Statistical analysis

Statistical analysis was performed using GraphPad Prism for Windows version 10.4.1. The viral titers were compared using multiple unpaired t test with log-transformed viral loads, with multiple comparisons corrected using the Benjamini, Krieger, and Yekutieli method.

References

1. World Health Organization. WHO information for the molecular detection of influenza viruses. Revision February 2021. Available at https://cdn.who.int/media/docs/default-source/influenza/molecular-detention-of-influenza-viruses/protocols_influenza_virus_detection_feb_2021.pdf?sfvrsn=df7d268a_5. Accessed on 11th April 2024 **2021**.
2. Yip CC, Chan WM, Ip JD, et al. Nanopore Sequencing Reveals Novel Targets for Detection and Surveillance of Human and Avian Influenza A Viruses. J Clin Microbiol **2020**; 58.
3. Katoh K, Standley DM. MAFFT multiple sequence alignment software version 7: improvements in performance and usability. Mol Biol Evol **2013**; 30:772-80.
4. Minh BQ, Schmidt HA, Chernomor O, et al. IQ-TREE 2: New Models and Efficient Methods for Phylogenetic Inference in the Genomic Era. Mol Biol Evol **2020**; 37:1530-4.
5. To KK, Chan WM, Ip JD, et al. Unique Clusters of Severe Acute Respiratory Syndrome Coronavirus 2 Causing a Large Coronavirus Disease 2019 Outbreak in Hong Kong. Clin Infect Dis **2021**; 73:137-42.
6. Katoh K, Misawa K, Kuma K, Miyata T. MAFFT: a novel method for rapid multiple sequence alignment based on fast Fourier transform. Nucleic Acids Res **2002**; 30:3059-66.
7. Okonechnikov K, Golosova O, Fursov M, team U. Unipro UGENE: a unified bioinformatics toolkit. Bioinformatics **2012**; 28:1166-7.
8. Hall TA. BioEdit: A User-Friendly Biological Sequence Alignment Editor and Analysis Program for Windows 95/98/NT. Nucl Acids Symp Ser **1999**; 41:95-8.
9. Jumper J, Evans R, Pritzel A, et al. Highly accurate protein structure prediction with AlphaFold. Nature **2021**; 596:583-9.

10. Evans R, O'Neill M, Pritzel A, et al. Protein complex prediction with AlphaFold-Multimer. biorxiv **2021**:2021.10. 04.463034.
11. Case DA, Cheatham TE, 3rd, Darden T, et al. The Amber biomolecular simulation programs. J Comput Chem **2005**; 26:1668-88.
12. Mänz B, Dornfeld D, Götz V, et al. Pandemic Influenza A Viruses Escape from Restriction by Human MxA through Adaptive Mutations in the Nucleoprotein. PLOS Pathogens **2013**; 9:e1003279.
13. Gray JJ, Moughon S, Wang C, et al. Protein-protein docking with simultaneous optimization of rigid-body displacement and side-chain conformations. J Mol Biol **2003**; 331:281-99.
14. Peiris M, Yuen KY, Leung CW, et al. Human infection with influenza H9N2. Lancet **1999**; 354:916-7.
15. Zhang X, Lam SJ, Ip JD, et al. Characterizing fitness and immune escape of SARS-CoV-2 EG.5 sublineage using elderly serum and nasal organoid. iScience **2024**; 27:109706.
16. Zhou J, Li C, Sachs N, et al. Differentiated human airway organoids to assess infectivity of emerging influenza virus. Proc Natl Acad Sci U S A **2018**; 115:6822-7.
